# Analysis of the Antennal Transcriptome and Insights into Olfactory Genes in *Hyphantria cunea* (Drury)

**DOI:** 10.1371/journal.pone.0164729

**Published:** 2016-10-14

**Authors:** Long-Wa Zhang, Ke Kang, Shi-Chang Jiang, Ya-Nan Zhang, Tian-Tian Wang, Jing Zhang, Long Sun, Yun-Qiu Yang, Chang-Chun Huang, Li-Ya Jiang, De-Gui Ding

**Affiliations:** 1 Anhui Provincial Key Laboratory of Microbial Control, School of Forestry & Landscape Architecture, Anhui Agricultural University, Hefei, 230036, China; 2 College of Life Sciences, Huaibei Normal University, Huaibei, 235000, China; 3 Forest Pests Control and Quarantine Bureau of Anhui Province, Hefei, 230001, China; Biogen Idec Inc, UNITED STATES

## Abstract

*Hyphantria cunea* (Drury) (Lepidoptera: Arctiidae) is an invasive insect pest which, in China, causes unprecedented damage and economic losses due to its extreme fecundity and wide host range, including forest and shade trees, and even crops. Compared to the better known lepidopteran species which use Type-I pheromones, little is known at the molecular level about the olfactory mechanisms of host location and mate choice in *H*. *cunea*, a species using Type-II lepidopteran pheromones. In the present study, the *H*. *cunea* antennal transcriptome was constructed by Illumina Hiseq 2500^TM^ sequencing, with the aim of discovering olfaction-related genes. We obtained 64,020,776 clean reads, and 59,243 unigenes from the analysis of the transcriptome, and the putative gene functions were annotated using gene ontology (GO) annotation. We further identified 124 putative chemosensory unigenes based on homology searches and phylogenetic analysis, including 30 odorant binding proteins (OBPs), 17 chemosensory proteins (CSPs), 52 odorant receptors (ORs), 14 ionotropic receptors (IRs), nine gustatory receptors (GRs) and two sensory neuron membrane proteins (SNMPs). We also found many conserved motif patterns of OBPs and CSPs using a MEME system. Moreover, we systematically analyzed expression patterns of OBPs and CSPs based on reverse transcription PCR and quantitative real time PCR (RT-qPCR) with RNA extracted from different tissues and life stages of both sexes in *H*. *cunea*. The antennae-biased expression may provide a deeper further understanding of olfactory processing in *H*. *cunea*. The first ever identification of olfactory genes in *H*. *cunea* may provide new leads for control of this major pest.

## Introduction

Olfaction plays a vital role in behaviors such as mating, foraging, and ovipositing for insects, especially Lepidoptera [1, 2]. Various odorants are sensed by insects using receptors on the antennae, and particularly, sensilla with a special hair-like structures [3]. The peripheral olfactory proteins involved in the reception of odorants in insects include odorant binding proteins (OBPs), chemosensory proteins (CSPs), odorant receptors (ORs), ionotropic receptors (IRs), gustatory receptors (GRs), and sensory neuron membrane proteins (SNMPs) [4–6]. In the periphery process of insect olfaction, firstly, external odorants enter into the chemosensilla and are captured by OBPs or CSPs, then the OBP or CSP bound odorants are transduction to ORs, triggering the transduction of chemical signals to electric signal [5, 7, 8]. GRs, IRs are another two receptors, which also participate in the chemreception. GRs are involved in contact chemoreception [9, 10]. IRs were more recently identified as a novel chemoreceptor family which evolved from ionotropic glutamate receptors (iGluRs) [6, 11, 12]. In addition, SNMPs are belong to the CD36 membrane proteins family that are located on dendrites and are crucial for pheromone recognition [11, 13].

The fall webworm, *Hyphantria cunea* (Drury) (Lepidoptera: Arctiidae), is a devastating invasive insect, which is widely distributed in North America, its region of origin [14]. *Hyphantria cunea* was first discovered in Dandong of Liaoning Province in China in 1979, and expanded its range rapidly to Hebei, Beijing, Shandong, Shaanxi, and Anhui provinces [14–18]. To date, *H*. *cunea* has caused unprecedented economic losses in China due to its shift from univoltinism to multivoltinism and extremely broad host range, devastating damage to forests, fruit trees, and even agricultural crops [15]. The fall webworm has been listed as one of the most important forest quarantine pests nationwide in China.

Moth sex pheromones are usually comprised of several components in specific ratios, and divided into two types, Type-I and Type-II according to the chemical functional groups [19, 20]. The pheromones of *H*. *cunea* are of Type-II. Although some research has been done on species using Type-I pheromones, few studies have focused on species secreting Type-II pheromones, which includes *H*. *cunea*. As an invasive species, sensitivities to and binding effects of plant volatiles may increase selectivity and adaptability to host plants, which may enhance the invasive capability of *H*. *cunea* and lead to more severe damage [21]. Although wide-ranging studies on the olfactory molecular mechanisms and identification of chemosensory genes have been reported for a number of lepidopteran species [22–28], this is not for *H*. *cunea*. Differentiation of sex pheromones in various moth species has occurred over many millennia, using diverse biosynthetic pathways involving different enzymes, substrates, and binding sites, resulting in the two major recognized pheromone types [29]. To date, little is known about the olfactory proteins repertoire species using Type-II pheromones. Did they evolve a novel receptors to perceive the Type-II sex pheromones, or were existing pheromone receptors were recruited for detection of the new ligands [29]? We assume that specific *H*. *cunea* pheromone-binding proteins (PBPs) are used binding its sex pheromone components. Thus, it is important to identify the olfactory genes in order to elucidate the molecular mechanisms of olfaction, and verify the existence of unique PBPs or other receptors in *H*. *cunea*. Chemical cues are also important for host location for parasitoid natural enemies of *H*. *cunea*. Among the many native natural enemies discovered to date [18, 30, 31], *Chouioia cunea* Yang (Hymenoptera: Eulophidae) was selected as a new biological control method against *H*. *cunea* [32, 33]. Because of the potential importance of *C*. *cunea* as an effective biocontrol agent against *H*. *cunea* and potential overlap in olfactory chemosensory ability, or "chemosphere", we compared our *H*. *cunea* OBPs and CSPs with previously published work on *C*. *cunea* in order to gain a better understanding of the possible olfactory mechanisms of an herbivore-natural enemy system.

In this study, we used the Illumina Hiseq 2500^TM^ platform to sequence the antennal transcriptome of *H*. *cunea*. After analyzing the transcriptome data, we identified 124 olfaction-related genes in total, including 30 OBPs, 17 CSPs, 52 ORs, 14 IRs, 9 GRs, and two SNMPs. In addition, the predicted protein sequences were compared with orthologs from moth species by building phylogenetic trees, and motif patterns of OBPs and CSPs were also constructed. On the basis of analyzing the antennal transcriptome, gene functional annotation was also obtained. Furthermore, OBPs and CSPs expression patterns in different tissues and development stages were determined using reverse transcription PCR (RT-PCR) and quantitative real time PCR (RT-qPCR). Lastly, we constructed phylogenetic trees of OBPs and CSPs based on our *H*. *cunea* data and previous published work on *C*. *cunea* to access the potential overlap in olfactory chemosensory ability.

## Materials and Methods

### Insect rearing and antennae collection

Pupae of *H*. *cunea* were collected from straws bundled around host trees (*Populus canadensis*) at Sixian, Anhui Province, China, and were maintained in plastic tubes. Tubes were buried in wet sand to provide high humidity, and were held at 25°C. Forest Pest Control Station of Anhui Province issued the permit for the field collection (by the director, Jun Fu). To eliminate the differences in each individual, the antennae from 60 newly emerged unmated moths (40 males and 20 females) were dissected, flash frozen in liquid nitrogen, and stored at -80°C until RNA extraction.

### RNA extraction and preparation of cDNA library

The stored antennae were ground and homogenized by vitreous Tissue-tearors (DEPC-water treated). Total RNA was extracted using TRIzol reagent (Invitrogen, USA) following the manufacturer’s instructions. RNA degradation and contamination was monitored on 1% agarose gels, and purity was checked using a NanoPhotometer^®^ spectrophotometer (IMPLEN, CA, USA). Illumina sequencing of the samples was performed at Novogene Co., Ltd., Beijing, China. Sequencing libraries were generated using NEBNext^®^ Ultra RNA Library Prep Kit for Illumina^®^ (New England Biolabs, USA) following the manufacturer’s recommendations. Briefly, mRNA was purified from total RNA using poly-T oligo-attached magnetic beads. First strand cDNA was synthesized using random hexamer primers and M-MLV Reverse Transcriptase (RNaseH). Second strand cDNA synthesis was subsequently performed using DNA Polymerase I and RNase H. Then, DNA fragments were treated for end-repairing, adenylation of 3’ ends and ligation of adaptors. The library fragments were purified with AMPure XP system (Beckman Coulter, CA, USA) to preferentially select cDNA fragments of 150~200 bp in length. Then, suitable fragments were enriched by PCR amplification.

### Transcriptome sequencing and assembly

The library preparations were sequenced on an Illumina Hiseq^TM^ 2500 platform and paired-end reads were generated. Clean reads were obtained by removing reads containing adapter, reads containing poly-N, and low quality reads from the raw reads. Transcriptome assembly was accomplished based on clean data with high quality using Trinity [34] to produce transcripts. Then the longest transcript of each single gene was selected as a unigene.

### Gene functional annotation

Unigenes obtained from antennae of *H*. *cunea* were identified by BLAST searches with annotation against the Nr database using an e-value cut-off of 10^−5^. The unigene sequences were also aligned to protein databases such as Swiss-Prot, Pfam, KOG/COG and KO to find the highest similarity to the given unigenes along with putative functional annotations. Blast2GO v2.5 [35] was used to get GO annotation, and GO enrichment analysis of the differentially expressed genes was implemented by the GOseqR packages based on Wallenius non-central hyper-geometric distribution [36]. The open reading frame (ORF) of each gene was determined using an ORF finder tool (http://www.ncbi.nlm.nih.gov/gorf/gorf.html). The signal peptide of the protein sequences was predicted using SignalP 4.0 [37]. The transmembrane domains of ORs, GRs, IRs and SNMPs were predicted by using TMHMM Server v. 2.0 (http://www.cbs.dtu.dk/services/TMHMM/).

### Phylogenetic analysis

Phylogenetic trees were built based on amino acid sequence alignment of the candidate OBPs, CSPs, ORs, GRs, IRs, and SNMPs from *H*. *cunea* and those of other insects species using ClustalX2.0 [38]. The OBP data set contained 30 identified sequences from *H*. *cunea*, 12 from *Agrotis ipsilon*, 43 from *Bombyx mori*, five from *Danaus plexippus*, 20 from *H*. *armigera*, 13 from *Helicoverpa assulta*, 14 from *Manduca sexta*, 24 from *Spodoptera exigua*, 20 from *Sesamia inferens*, and 38 from *Spodoptera litura*. The CSP data set contained 17 sequences from *H*. *cunea*, eight from *A*. *ipsilon*, 16 from *B*. *mori*, 10 from *H*. *armigera*, 17 from *S*. *exigua*, and 20 from *S*. *inferens*. The OR data set contained 52 sequences from *H*. *cunea*, 62 from *B*. *mori*, 11 from *H*. *armigera*, 35 from *S*. *inferens*, one from *Operophtera brumata*, and one from *Agrotis segetum*. The GR data set contained nine sequences from *H*. *cunea*, one from *A*. *ipsilon*, 29 from *B*. *mori*, three from *D*. *plexippus*, three from *H*. *armigera*, 18 from *H*. *assulta*, one from *M*. *sexta*, and six from *S*. *exigua*. The IR data set contained 14sequences from *H*. *cunea*, 10 from *B*. *mori*, 14 from *Cydia pomonella*, 10 from *Dendrolimus houi*, 9 from *Dendrolimus kikuchii*, 18 from *Dorsophila melanogaster*, and two from *S*. *inferens*. The SNMP data set contained two sequences from *H*. *cunea*, two from *A*. *ipsilon*, one from *B*. *mori*, two from *H*. *armigera*, one from *H*. *assulta*, two from *H*. *virescens*, two from *M*. *sexta*, three from *S*. *exigua*, two from *S*. *inferens*, and three from *S*. *litura*. Unrooted phylogenetic trees were constructed by the neighbor-joining method with Poisson correction of genetic distances in MEGA5.0 [39] software. Node support was generated from 1,000 bootstrap pseudo replications of the data.

### Motif analysis of OBPs and CSPs

In order to find the potential conversed motif, we compared the motifs-pattern of OBPs and CSPs in different families of Lepidoptera. A total of 76 OBPs and 43 CSPs from *H*. *cunea*, *B*. *mori*, and *H*. *armigera* were used for motif discovery and pattern analysis. All the OBP and CSP sequences used in this study were translated to amino acid sequences. The MEME (version 4.11.1) online software (http://meme-suite.org/tools/meme), which has been widely used for discovery of protein motifs [7, 40–42], was used to discover and analyze the motifs in this analysis. The parameter settings used for motif discovery were as follows: minimum width = 6, maximum width = 10, and the maximum number of motifs = 8.

### Tissue expression analysis of OBPs and CSPs

The expression patterns of OBPs and CSPs in different tissues (antennae, thoraces, abdomens, legs, wings) and life stages (pupae of both sexes and larvae) were analyzed by RT-PCR. Fifty male and female antennae, 10 whole insect body without antennae, thoraces, abdomens, legs, wings, and 10 pupae of both sexes and 10 larvae were collected, and frozen in liquid nitrogen for RT-PCR. Total RNA from different tissues was extracted as described above, including three replications of samples. PrimeScript^®^ RT reagent Kit with gDNA Eraser (Perfect Real Time, Takara, Dalian, China) was used for reverse transcription in order to remove residual trace amounts of genomic DNA. The cDNA (20 ng) was used as a template in RT-PCR. Primers were designed with the Primer Premier5 software (PREMIER Biosoft International, CA, USA). *EF1-a*–*H*. *cunea* voucher W72 elongation factor 1 alpha gene–was used as a reference gene. The cDNA template was replaced by RNase-free water in the negative control. PCR reaction was carried out under the conditions of 94°C for 30s, 52°C for 30s, 72°C for 15s using 2xEs Taq Master Mix (CWBIO, Beijing, China) in 30 cycles. PCR products were run on a 1% agarose gel.

The expression patterns of OBPs and CSPs in different tissues (male antennae, female antennae, legs, wings) and life stages (pupae of both sexes and larvae) were analyzed by RT-qPCR. Twenty male and female antennae, 20 legs, 20 wings, and 10 pupae of both sexes and 10 larvae were collected, and frozen in liquid nitrogen for RT-qPCR. cDNAs from antennae and other tissues were synthesized as described above. The equal amount of cDNA (2.5 ng) was used as a template in RT-qPCR. Primers were designed with the Beacon Designer 7.9 software (PREMIER Biosoft International, CA, USA). *HyphEF1-a* (elongation factor 1 alpha gene) and *HyphGAPDH* were used as the reference genes. The cDNA template was replaced by RNase-free water in the negative control. The RT-qPCR was performed on a CFX96 Detection System (Bio-rad, Hercules, CA, USA) using a mixture of 25μL reaction: 12.5μLSYBR^®^ Premix Ex Taq II (Tli RNaseH Plus) (Takara, Dalian, China), 1μL of each primer (10μM), 2.0μL of template cDNA, and 8.5μL of sterilized ultrapure H_2_O. The RT-qPCR reaction was carried out under the conditions of 95°C for 30s, followed by 40 cycles of 95°C for 5s and 60°C for 30s, then the melting curve was measured. Each sample included three biological replications which measured in three technique replications. The RT-qPCR results were analyzed using the CFX96 analysis software, and the expression levels of above genes were calculated relative to two reference genes using the Q-gene method [43, 44]. Data of relative expression levels from various samples were subjected to ANOVA (one-way analysis of variance), followed by Duncan's new multiple range test using the SPSS 22.0 software (SPSS Inc., Chicago, IL, USA).

## Results

### Unigene assembly and transcriptome sequencing

A total of 65,177,438 raw reads were obtained from an Illumina Hiseq 2500 platform (Table 1). After removing adaptors and low quality reads, 64,020,776 clean reads were acquired with a Q20 percentage of 96.03%, which were assembled into 78,131 transcripts with a mean length of 1123 bp and an N50 length of 2520 bp. 59,243 unigenes were selected from the above transcripts with a mean length of 829 bp and an N50 length of 1803 bp. 35,976 unigenes were longer than 300 bp which accounted for 60.73% of all unigenes (S1 Fig).

**Table 1:** Summary of the antennal transcriptome of *H*. *cunea*.

Statistics Project	Number
Total raw reads	65177438
Total clean reads	64020776
Clean bases	8G
Q20 percentage	96.03%
Q30 percentage	92.28%
GC percentage	41.23%
Transcripts	78131
Mean length of transcripts	1123
N50 of transcripts	2520
Unigenes	59243
Mean length of unigenes	829
N50 of unigenes	1803

### Homology analysis and gene functional annotation

The functional annotation of unigenes was performed by a BLAST homology search against the protein databases. 15,242 (25.72%) unigenes were annotated in the Nr database. As a result, 91.30% of annotated unigenes had more than 60% similarity with known proteins (S2A Fig). The e-value distribution showed that 64.90% of the annotated unigenes had strong homology (e-value < 1e-45), whereas 13.90% of the unigenes had low homology (1e-15<e-value<1e-5) (S2B Fig). The species classification showed that the best match was *B*. *mori*, representing 48.10%, followed by *D*. *plexippus* (29.50%), *Papilio xuthus* (2%), and *H*. *armigera* (1.7%) (S2C Fig). The high similarity between *H*. *cunea* and *B*. *mori* maybe due to the genome of *B*. *mori* having been reported [45, 46] with numerous proteins in the NCBI protein database used for homology analyzing.

GO annotation was obtained using the program Blast2GO against the Nr database. A total of 12,565 unigenes were assigned to three main GO classes among all 59,243 unigenes. Specifically: these included genes for biological processes (34,685), cellular components (22,506), and molecular function (15,726) (S3 Fig). In the molecular function category, binding (7,161) and catalytic activity (5,275) were two major terms of antennal gene expression. In the biological processes, cellular processes (7,295), metabolic processes (6,606), and single-organism processes (5,716) were the most abundant. Cell (4,446) and cell parts (4,446) were enriched in the same level of cellular component, followed by organelle (2946), macromolecular complex (2661) and membrane (2523).

After a total of 5,781 unigenes were annotated in the KO database, we acquired a KEGG pathway classification for the *H*. *cunea* antennal transcriptome. Five subcategories of KEGG pathway were as follows: cellular processes (A), environmental information processing (B), genetic information processing (C), metabolism (D), and organisimal systems (E) (S4 Fig). Signal transduction (698) was the highest term in the environmental information processing subcategory, which indicated the strong association with odorant binding and transduction of the antennal tissue. In addition, genes associated with biodegradation and metabolism of xenobiotics (130) were identified; these are likely involved in odorant degradation in olfactory processes.

### Identification of putative odorant-binding proteins

**Fig 1:**
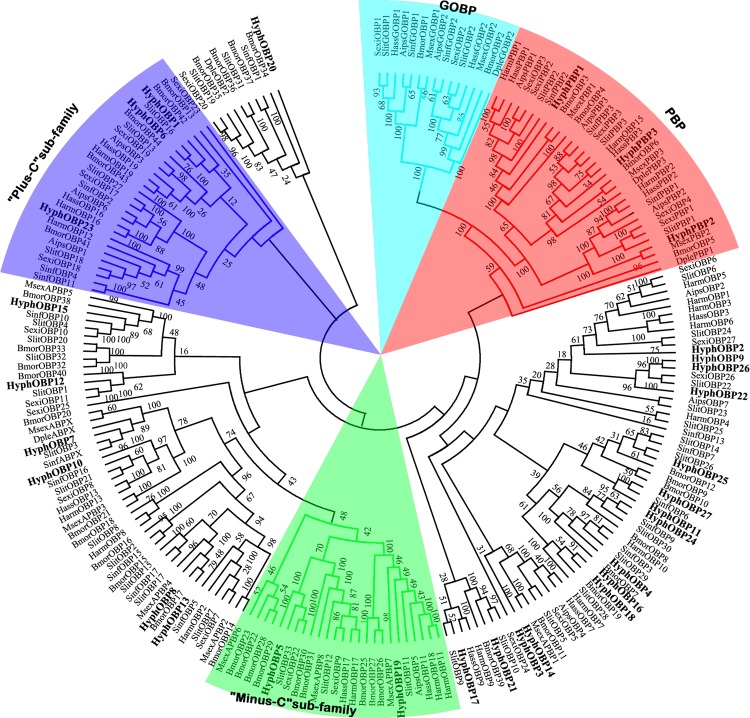
Phylogenetic tree of putative odorant binding protein (OBP) genes from *H*. *cunea* and other lepidopteran insects. The tree was constructed with MEGA5.0, which was based on amino acid sequence alignments by using the ClustalX2.0. Aips: *A*. *ipsilon*; Bmor: *B*. *mori*; Dple: *D*. *plexippus*; Harm: *H*. *armigera*; Hass: *H*. *assulta*; Msex: *M*. *sexta*; Sexi: *S*. *exigua*; Sinf: *S*. *inferens*; Slit: *S*. *litura*.

Analysis of the *H*. *cunea* antennal transcriptome identified 30 putative OBPs, including 3 PBPs (Table 2). The signal peptide prediction showed that 26 unigenes had a complete ORF (Table 3). Most OBPs had a low similarity to known lepidopteran OBPs, possibly due to the relatively low conservation among different families. OBPs can be generally divided into different subclasses according to the number of conserved cysteines, including Classic OBPs, Plus-C OBPs and Minus-C OBPs [47]. We identified 15 classic OBPs using multiple amino acid sequence alignments, which were matched up with the six-cysteines pattern C1-X_25-30_-C2-X_3_-C3-X_36-42_-C4-X_8-14_-C5-X_8_-C6 (where X stands for any amino acid) proposed by Xu et al. [7] (S5 Fig). A phylogenetic tree was constructed based on the neighbor-joining method (Fig 1). HyphOBP1, 6, and 23 clustered with the Plus-C subfamily, whereas the Minus-C subfamily contained HyphOBP5 and 19. General OBPs clustered together with PBPs, including HyphPBP1, 2, and 3, which all belong to the classic OBPs.

**Table 2:** Summary of candidate genes from the antennal transcriptome of *H*. *cunea*.

Candidate genes	Number
Odorant binding proteins	30
Odorant receptors	52
Chemosensory proteins	17
Gustatory receptors	9
Ionotropic receptors	14
Sensory neuron membrane proteins	2

**Table 3:** Blastx matches of *H*. *cunea* putative OBP genes.

Gene Name	ORF Length (bp)	Complete ORF	Signal Peptide	FPKM value	Best Blastx Match			
					Species	Acc.number	e-value	Identity (%)
OBP1	458	NO	1–18	1.32	*Spodoptera litura*	ALD65890.1	2e-08	28
OBP2	441	YES	1–20	39148.54	*Helicoverpa armigera*	AEB54581.1	2e-49	58
OBP3	391	NO	1–23	1.95	*Spodoptera litura*	AKI87966.1	4e-17	36
OBP4	438	YES	1–17	10.3	*Sesamia inferens*	AGS36745.1	8e-60	79
OBP5	507	YES	1–29	2.25	*Helicoverpa assulta*	AGC92792.1	6e-14	34
OBP6	504	YES	1–16	82.37	*Spodoptera litura*	ALD65890.1	1e-24	35
OBP7	420	YES	1–25	1521.66	*Spodoptera litura*	AKI87964.1	4e-60	84
OBP8	438	YES	1–24	1175.52	*Cnaphalocrocis medinalis*	AFG72998.1	9e-74	79
OBP9	462	YES	1–21	2.13	*Helicoverpa armigera*	AEB54581.1	4e-43	52
OBP10	426	YES	1–18	5457.53	*Sesamia inferens*	AGS36756.1	2e-59	89
OBP11	291	YES	0	4.14	*Sesamia inferens*	AGS36748.1	7e-29	46
OBP12	684	YES	1–19	355.49	*Spodoptera litura*	AKI87962.1	4e-56	69
OBP13	429	YES	1–21	51.43	*Helicoverpa assulta*	AEX07275.1	1e-60	78
OBP14	453	YES	1–25	44.62	*Spodoptera exigua*	ADY17883.1	7e-16	37
OBP15	489	YES	1–19	3438.76	*Sesamia inferens*	AGS36751.1	2e-52	61
OBP16	459	YES	1–20	15.6	*Spodoptera exigua*	AGP03460.1	4e-44	52
OBP17	591	YES	1–21	35.88	*Spodoptera litura*	ALD65883.1	2e-70	81
OBP18	447	YES	1–18	7.53	*Spodoptera exigua*	AGP03460.1	1e-36	47
OBP19	414	YES	1–16	32.43	*Helicoverpa armigera*	AFI57167.1	7e-43	64
OBP20	399	YES	1–20	11.89	*Helicoverpa armigera*	AEB54582.1	2e-05	29
OBP21	552	YES	1–20	9.66	*Dendrolimus houi*	AII00978.1	1e-113	91
OBP22	450	YES	1–22	21.58	*Helicoverpa assulta*	AEX07270.1	8e-34	45
OBP23	564	YES	1–17	6.18	*Agrotis ipsilon*	AGR39564.1	2e-50	51
OBP24	447	YES	1–19	70.21	*Sesamia inferens*	AGS36750.1	1e-42	68
OBP25	462	YES	1–23	13.91	*Spodoptera exigua*	AGP03457.1	1e-69	66
OBP26	357	NO	1–21	0.84	*Helicoverpa assulta*	AEX07271.1	4e-35	50
OBP27	520	NO	1–13	0.97	*Sesamia inferens*	AGS36748.1	2e-33	47
PBP1	492	YES	1–19	15890.73	*Helicoverpa armigera*	AEB54585.1	1e-77	70
PBP2	507	YES	1–24	5157.22	*Manduca sexta*	AAF16711.1	7e-70	63
PBP3	525	YES	0	1140.45	*Spodoptera exigua*	ACY78413.1	5e-68	66

### Identification of putative chemosensory proteins

**Fig 2:**
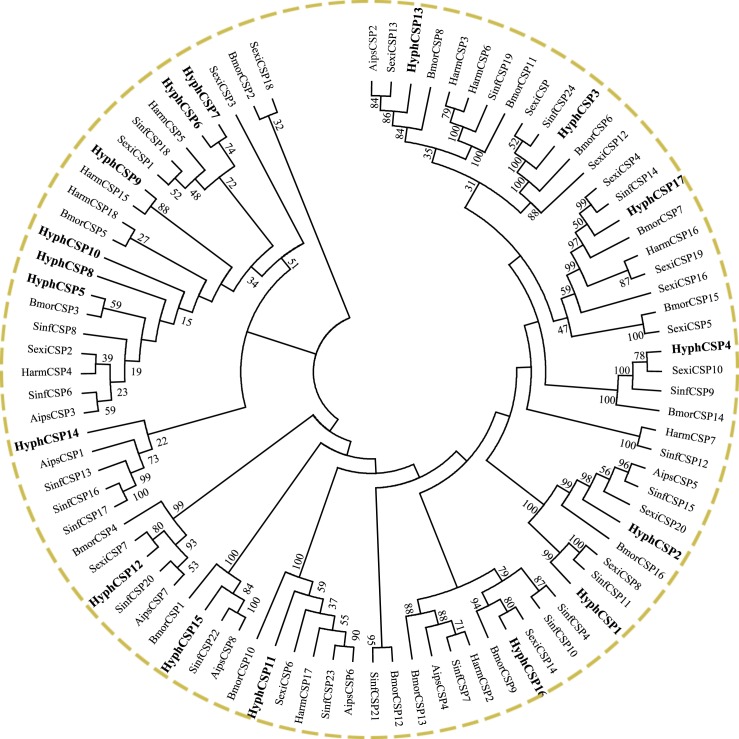
Phylogenetic tree of putative chemosensory protein (CSP) genes from *H*. *cunea* and other lepidopteran insects. Aips: *A*. *ipsilon*; Bmor: *B*. *mori*; Harm: *H*. *armigera*; Sexi: *S*. *exigua*; Sinf: *S*. *inferens*.

Seventeen putative CSPs were identified in the *H*. *cunea* antennal transcriptome (Table 2), verified by the four-cysteines pattern C1-X_6_-C2-X_18_-C3-X_2_-C4 (S6 Fig). Among these sequences, 16 had a complete ORF with a predicted signal peptide. Almost 90% of the CSPs (15) had more than 70% similarity with other species’ CSPs, much higher than the sequence similarities of the OBPs (26.7%) (Table 4). This indicated that the CSPs are more highly conserved than OBPs. The CSPs were scattered in different branches of the phylogenetic tree, except HyphCSP6 and HyphCSP7, which clustered in the same subfield (Fig 2).

**Table 4:** Blastx matches of *H*. *cunea* putative CSP genes.

Gene Name	ORF Length (bp)	Complete ORF	Signal Peptide	FPKM value	Best Blastx Match			
					Species	Acc.number	e-value	Identity (%)
CSP1	324	YES	1–17	1.8	*Sesamia inferens*	AGY49260.1	2e-36	85
CSP2	324	YES	1–18	26.13	*Agrotis ipsilon*	AGR39575.1	1e-61	90
CSP3	369	YES	1–16	4.17	*Helicoverpa armigera*	AFR92094.1	9e-70	83
CSP4	372	YES	1–19	1.31	*Sesamia inferens*	AGY49258.1	1e-44	94
CSP5	384	YES	1–18	49.16	*Heliothis virescens*	AAV34686.1	1e-51	79
CSP6	381	YES	1–18	290.29	*Sesamia inferens*	AGY49267.1	2e-60	74
CSP7	378	YES	1–18	4.49	*Heliothis virescens*	AAV34686.1	1e-51	79
CSP8	378	YES	1–18	5.2	*Spodoptera exigua*	ABM67689.1	2e-58	74
CSP9	378	YES	1–16	1121.78	*Helicoverpa armigera*	AGH20053.1	8e-51	74
CSP10	387	YES	1–18	28681.98	*Spodoptera exigua*	ABM67689.1	9e-59	73
CSP11	408	YES	0	638.97	*Helicoverpa armigera*	AGH20055.1	7e-60	86
CSP12	384	YES	1–16	2360.09	*Helicoverpa assulta*	ABB91378.1	3e-63	83
CSP13	372	YES	1–16	3.02	*Agrotis ipsilon*	AGR39572.1	4e-57	72
CSP14	387	YES	1–18	10.7	*Helicoverpa armigera*	AFR92095.1	5e-47	58
CSP15	372	YES	1–18	342.15	*Sesamia inferens*	AGY49271.1	3e-50	71
CSP16	870	YES	1–16	92.91	*Helicoverpa armigera*	AIW65104.1	2e-102	70
CSP17	331	NO	1–18	7.33	*Spodoptera exigua*	AKT26481.1	9e-42	63

### Identification of putative odorant receptors and gustatory receptors

**Fig 3:**
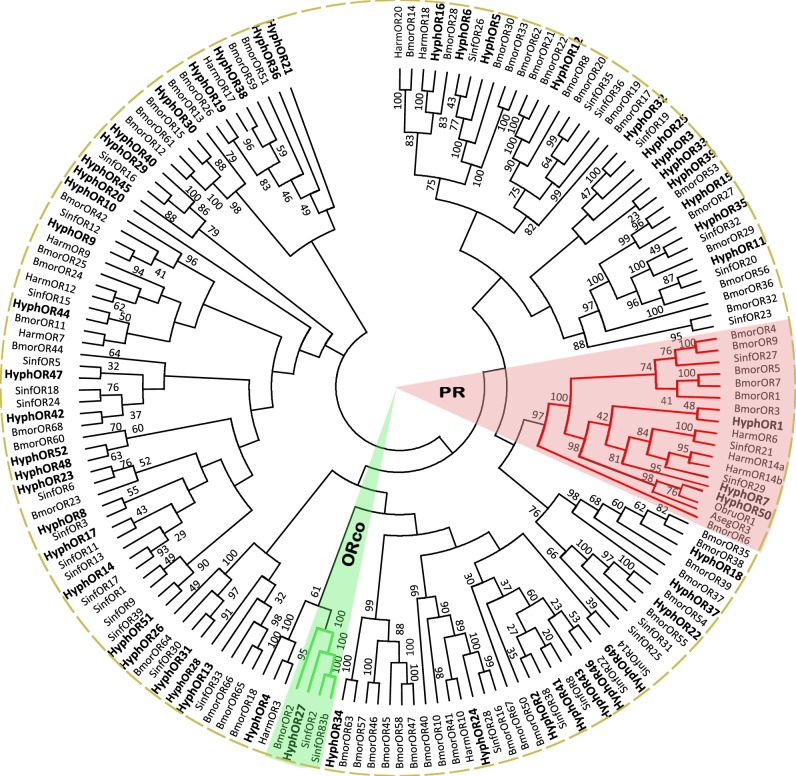
Phylogenetic tree of putative odorant receptor (OR) genes from *H*. *cunea* and other lepidopteran insects. Bmor: *B*. *mori*; Harm: *H*. *armigera*; Sinf: *S*. *inferens*; Obru: *O*. *brumata*; Aseg: *A*. *segetum*.

We identified 52 putative ORs by analyzing the antennal transcriptome (Table 2). The TMHMM prediction showed that five unigenes (HyphOR9, 12, 21, 27 and 34) had seven-transmembrane domains, and 42 sequences had a full-length ORF (Table 5). Fifty-two sequences showing multiple amino acid alignment with ORs from *B*. *mori*, *H*. *armigera*, *S*. *inferens*, *O*. *brumata*, and *A*. *segetum* were used to construct a phylogenetic tree (Fig 3). HyphOR27 clustered with the lepidopteran ORco (olfactory receptor coreceptor) family and had a high degree of similarity with these ORs. The lepidopteran PR family was also detected, and HyphOR1, 7, 50 belonged to this family. In addition, HyphOR50 was clustered with ObruOR1 and AsegOR3, which had a high orthology.

**Table 5:** Blastx matches of *H*. *cunea* putative OR genes.

Gene Name	ORF Length (bp)	Complete ORF	Transmembrane Domain	FPKM value	Best Blastx Match			
					Species	Acc.number	e-value	Identity (%)
OR1	636	YES	0	2.88	*Helicoverpa armigera*	AIG51897.1	2e-78	55
OR2	339	YES	1	1.39	*Dendrolimus houi*	AII01061.1	3e-13	71
OR3	1131	YES	5	1.75	*Bombyx mori*	NP_001091791.1	1e-17	42
OR4	891	YES	5	2.95	*Helicoverpa armigera*	AGK89999.1	1e-129	61
OR5	1215	YES	6	78.14	*Planotortrix octo*	AJF23783.1	2e-131	49
OR6	1191	YES	5	67.38	*Ctenopseustis herana*	AIT69871.1	1e-125	50
OR7	720	YES	3	1.14	*Helicoverpa assulta*	AGK90014.1	1e-84	59
OR8	450	YES	1	5.66	*Helicoverpa armigera*	AIZ00995.1	8e-137	59
OR9	1161	YES	7	7.86	*Dendrolimus kikuchii*	AII01102.1	5e-166	64
OR10	1158	YES	5	4.94	*Dendrolimus kikuchii*	AII01102.1	9e-127	56
OR11	1209	YES	5	5.1	*Helicoverpa armigera*	AIG51887.1	0.0	81
OR12	1203	YES	7	4.33	*Bombyx mori*	NP_001166613.1	8e-165	64
OR13	1272	YES	4	6.47	*Danaus plexippus*	EHJ75140.1	4e-53	56
OR14	609	YES	2	3.76	*Helicoverpa armigera*	AIG51888.1	2e-97	77
OR15	1098	YES	5	12.12	*Dendrolimus kikuchii*	AII01090.1	3e-132	58
OR16	1197	YES	3	4.51	*Helicoverpa assulta*	ADN03364.1	0.0	73
OR17	648	YES	2	23.2	*Dendrolimus kikuchii*	AII01083.1	8e-66	46
OR18	1242	YES	6	6.03	*Helicoverpa armigera*	AIG51898.1	0.0	64
OR19	915	YES	5	21.05	*Helicoverpa armigera*	AIG51873.1	1e-118	66
OR20	1347	YES	4	6.2	*Spodoptera litura*	AGG08878.1	6e-161	57
OR21	1242	YES	7	12.81	*Spodoptera exigua*	AEF32141.1	0.0	70
OR22	1161	YES	8	6.14	*Dendrolimus kikuchii*	AII01092.1	7e-137	59
OR23	281	NO	0	1.54	*Bombyx mori*	BAH66328.1	3e-16	64
OR24	1164	YES	6	10.5	*Bombyx mori*	NP_001104832.2	2e-146	54
OR25	604	NO	4	10.39	*Helicoverpa armigera*	AIG51890.1	6e-55	45
OR26	636	YES	0	11.8	*Helicoverpa assulta*	AJD81578.1	1e-90	63
OR27	1422	YES	7	489.83	*Heliothis viriplaca*	AFI25169.1	0.0	93
OR28	1191	YES	6	4.42	*Dendrolimus houi*	AII01045.1	7e-113	48
OR29	957	YES	4	11.96	*Dendrolimus houi*	AII01055.1	2e-58	51
OR30	1152	YES	6	4.95	*Bombyx mori*	NP_001091789.1	5e-117	53
OR31	1101	YES	4	21.41	*Dendrolimus kikuchii*	AII01083.1	6e-163	61
OR32	1359	YES	6	82.39	*Helicoverpa armigera*	AIG51892.1	0.0	72
OR33	609	YES	3	2.35	*Agrotis segetum*	AGS41446.1	7e-20	32
OR34	1011	YES	7	7	*Helicoverpa armigera*	AIG51889.1	1e-144	72
OR35	1188	YES	5	28.8	*Helicoverpa armigera*	AIG51879.1	0.0	75
OR36	1080	YES	4	11.88	*Spodoptera litura*	AGG08876.1	3e-140	65
OR37	1251	YES	4	3.49	*Ostrinia furnacalis*	BAR43458.1	8e-165	62
OR38	1020	YES	4	7.58	*Helicoverpa assulta*	AGK90020.1	9e-130	65
OR39	1455	YES	0	7.17	*Ostrinia furnacalis*	BAR43469.1	0.0	76
OR40	505	NO	1	1.89	*Ostrinia furnacalis*	BAR43481.1	3e-26	37
OR41	284	NO	0	1.08	*Danaus plexippus*	EHJ78030.1	3e-50	82
OR42	294	YES	1	1.41	*Bombyx mori*	NP_001166607.1	1e-50	83
OR43	330	NO	2	1.73	*Helicoverpa armigera*	ACF32962.1	2e-62	87
OR44	435	NO	1	0.86	*Helicoverpa assulta*	AGK90015.1	2e-61	77
OR45	1215	YES	6	2.23	*Ostrinia furnacalis*	BAR43494.1	4e-140	50
OR46	343	NO	0	1.62	*Danaus plexippus*	EHJ78030.1	2e-47	65
OR47	396	NO	1	0.84	*Spodoptera litura*	AGG08877.1	7e-69	79
OR48	318	YES	1	1.02	*Helicoverpa assulta*	AGK90015.1	9e-61	72
OR49	477	YES	3	1.3	*Bombyx mori*	XP_012545317.1	1e-52	53
OR50	1392	YES	5	390.73	*Spodoptera exigua*	AGH58120.1	2e-177	62
OR51	669	NO	4	1	*Helicoverpa armigera*	ACC63240.1	1e-85	55
OR52	708	NO	4	2.23	*Helicoverpa armigera*	AIG51888.1	1e-129	81

**Fig 4:**
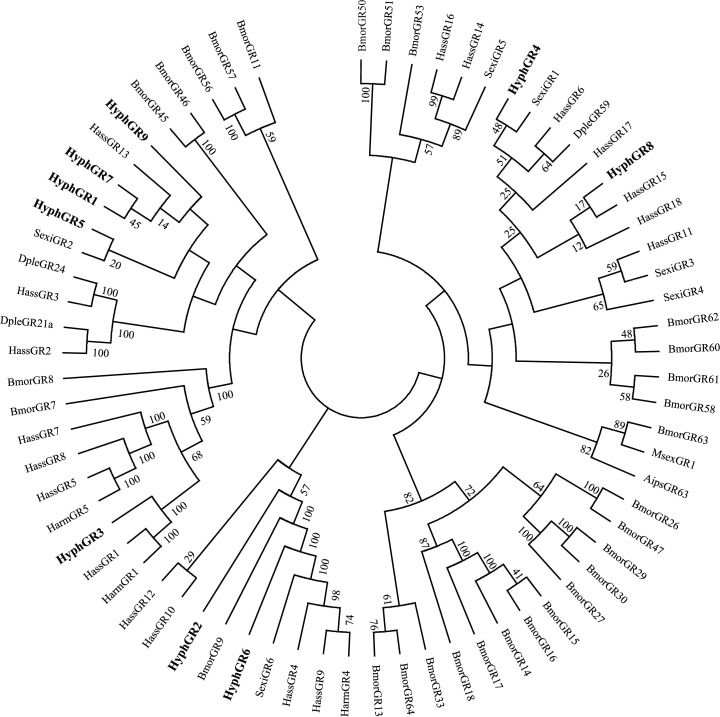
Phylogenetic tree of putative gustatory receptor (GR) genes from *H*. *cunea* and other lepidopteran insects. Aips: *A*. *ipsilon*; Bmor: *B*. *mori*; Dple: *D*. *plexippus*; Harm: *H*. *armigera*; Hass: *H*. *assulta*; Msex: *M*. *sexta*; Sexi: *S*. *exigua*.

Nine putative GRs were discovered and four of them had a complete ORF (HyphGR1, 2, 3, 7) (Tables 2 and 6). The prediction showed that HyphGR7 had none transmembrane domain (Table 6). The phylogenetic tree of the GRs showed that HyphGR1 and HyphGR7 clustered into the same branch, and HyphGR3 had a complete similarity (100%) with HassGR1 and HarmGR1 (Fig 4).

**Table 6:** Blastx matches of *H*. *cunea* putative GR and IR genes.

Gene Name	ORF Length (bp)	Complete ORF	Transmembrane Domain	FPKM value	Best Blastx Match			
					Species	Acc.number	e-value	Identity (%)
GR1	522	YES	2	1.31	*Helicoverpa assulta*	AJD81608.1	5e-32	49
GR2	1131	YES	5	1.75	*Bombyx mori*	BAS18817.1	2e-17	39
GR3	1335	YES	8	1.65	*Helicoverpa armigera*	AIG51912.1	5e-135	74
GR4	474	NO	2	0.72	*Helicoverpa armigera*	AGA04648.1	5e-68	81
GR5	395	NO	3	1.02	*Helicoverpa armigera*	AGK90011.1	1e-46	78
GR6	330	NO	2	1.73	*Helicoverpa armigera*	AGA04648.1	5e-62	87
GR7	294	YES	0	5.15	*Bombyx mori*	DAA06394.1	6e-04	36
GR8	243	NO	1	2	*Helicoverpa assulta*	AJD81596.1	2e-24	90
GR9	777	NO	5	1.17	*Helicoverpa assulta*	AJD81606.1	4e-17	38
IR1	475	NO	0	2.97	*Spodoptera littoralis*	ADR64681.1	5e-31	55
IR2	636	NO	3	1.5	*Helicoverpa armigera*	AIG51919.1	4e-81	68
IR3	459	YES	1	1.59	*Helicoverpa armigera*	AIG51922.1	8e-96	82
IR4	1395	YES	3	3.78	*Spodoptera littoralis*	ADR64681.1	0.0	74
IR5	1908	YES	3	5.65	*Spodoptera littoralis*	ADR64688.1	0.0	59
IR6	1908	YES	3	3.08	*Spodoptera littoralis*	ADR64689.1	0.0	79
IR7	1803	YES	3	14.06	*Spodoptera littoralis*	ADR64681.1	0.0	65
IR8	1671	YES	2	6.43	*Spodoptera littoralis*	ADR64678.1	0.0	78
IR9	1263	YES	3	22.04	*Sesamia inferens*	AGY49253.1	0.0	75
IR10	366	NO	0	1.29	*Helicoverpa armigera*	AIG51919.1	4e-56	72
IR11	1875	YES	3	4.75	*Spodoptera littoralis*	ADR64684.1	0.0	81
IR12	2763	YES	3	85.38	*Helicoverpa assulta*	AJD81628.1	0.0	93
IR13	1860	YES	5	46.6	*Spodoptera littoralis*	ADR64683.1	0.0	64
IR14	2697	YES	4	49	*Cydia pomonella*	AFC91764.1	0.0	78

### Identification of putative ionotropic receptors

**Fig 5:**
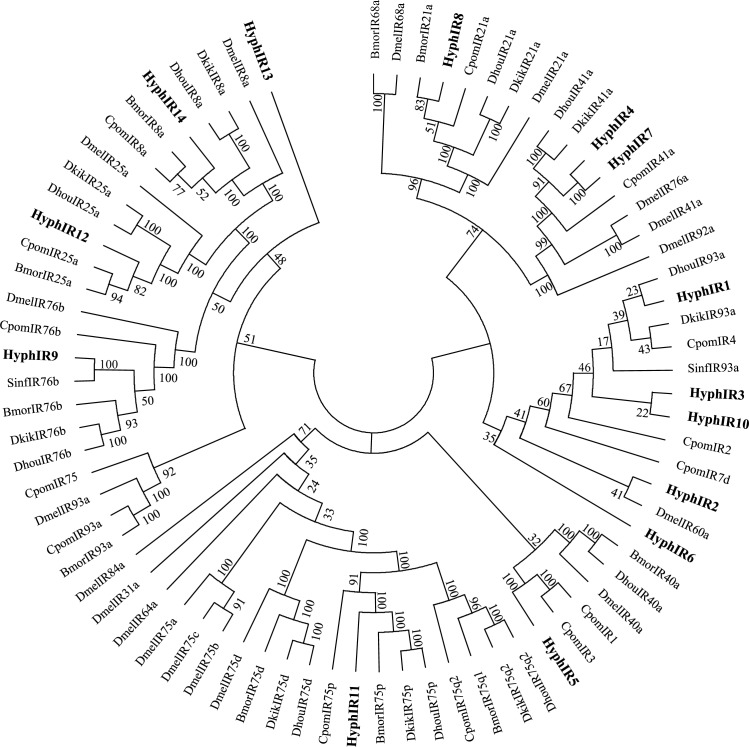
Phylogenetic tree of putative ionotropic receptor (IR) genes from *H*.*cunea* and other lepidopteran insects. Bmor: *B*. *mori*; Cpom: *C*. *pomonella*; Dhou: *D*. *houi*; Dkik: *D*. *kikuchii*; Dmel: *D*. *melanogaster*; Sinf: *S*. *inferens*.

Transcriptome assembly and analysis led to the identification of 14putative IRs (Table 2). 11 of the 14 sequences had a complete ORF, with HyphIR1, 2 and10 being the exceptions (Table 6). And two IRs (HyphIR1 and 10) had none transmembrane domain (Table 6). In the IR phylogenetic tree, 14 IR sequences were distributed in differential subclades. HyphIR4 and HyphIR7 may belong to the IR41a clade and had 100% orthology with each other. HyphIR9 clustered with the IR76b sequences from other insects, and showed homology with SinfIR76b, which may be characterized as HyphIR76b. The same situation occurred with HyphIR8 and HyphIR11, which could be identified as a member of the IR21a and IR75p subgroup, respectively. In addition, HyphIR14 was found in the high conserved IR8a subfamily, while HyphIR12 was a member of IR25a subclade (Fig 5).

### Identification of putative sensory neuron membrane proteins

**Fig 6:**
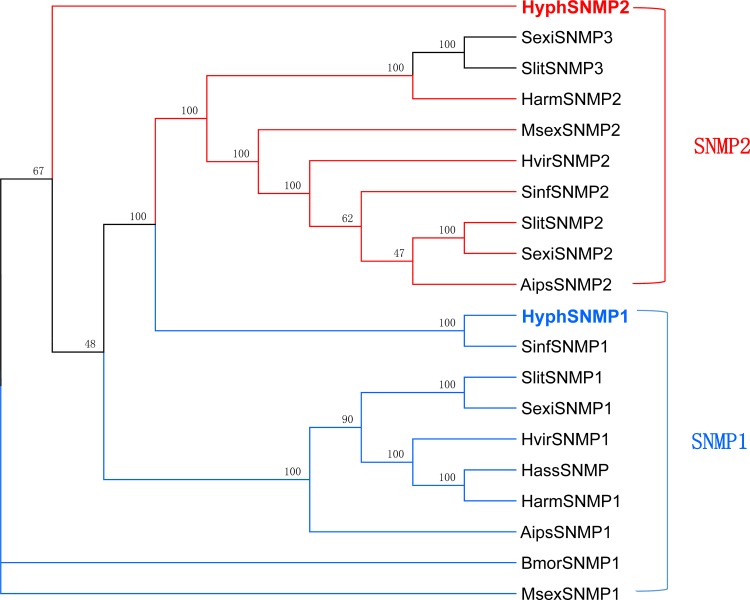
Phylogenetic tree of putative sensory neuron membrane protein (SNMP) genes from *H*. *cunea* and other lepidopteran insects. Aips: *A*. *ipsilon*; Bmor: *B*. *mori*; Harm: *H*. *armigera*; Hass: *H*. *assulta*; Hvir: *H*. *virescens*; Msex: *M*. *sexta*; Sexi: *S*. *exigua*; Sinf: *S*. *inferens*; Slit: *S*. *litura*.

Two SNMPs (SNMP1 and SNMP2) were detected from our transcriptome (Table 2). SNMP2 was presumed to have a full-length ORF (Table 7). In the phylogenetic tree, HyphSNMP1 clustered with SinfSNMP1 belonging to the SNMP1 family. HyphSNMP2 formed a unique branch in the SNMP2 family (Fig 6).

**Table 7:** Blastx matches of *H*. *cunea* putative SNMP genes.

Gene Name	ORF Length (bp)	Complete ORF	Transmembrane Domain	FPKM value	Best Blastx Match			
					Species	Acc.number	e-value	Identity (%)
SNMP1	257	NO	0	1.38	*Bombyx mori*	XP_012550444.1	1e-25	52
SNMP2	1578	YES	2	254.74	*Heliothis virescens*	Q9U1G3.1	0.0	76

### Motif pattern analysis of *H*. *cunea* OBPs and CSPs

**Fig 7:**
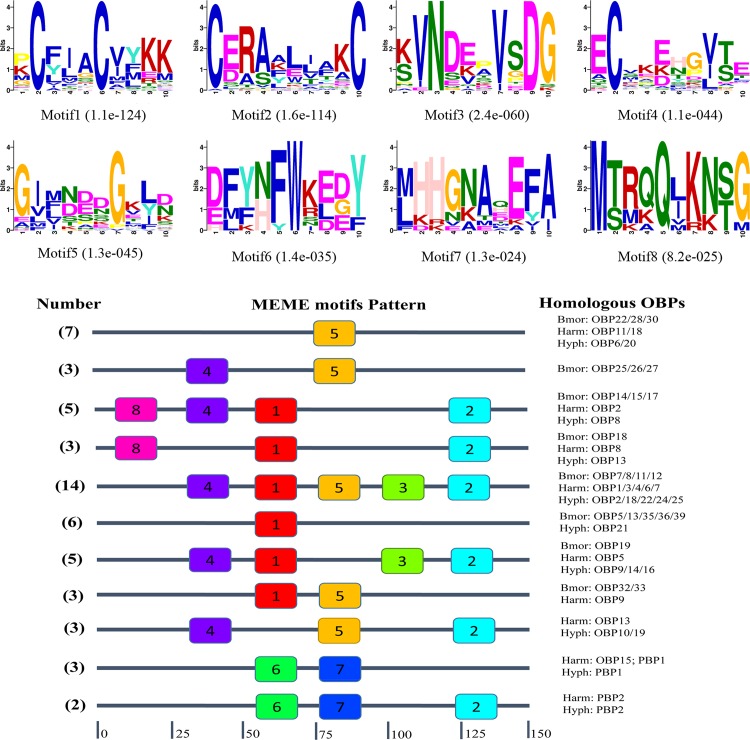
Motif analysis of OBPs in *H*. *cunea*. The upper parts listed the eight motifs discovered in 76 OBPs. The lower parts indicate approximate locations of each motif on the protein sequence. The numbers in the colored boxes correspond to the numbered motifs in the upper part of the figure. The small number represents high conservation. The numbers on the bottom show the approximate locations of each motif on the protein sequence, starting from the N-terminal. This figure just listed 11 relatively common motif patterns including 54 OBPs.

The purpose of conserved motifs analyses are an important step to better understand the functional domains and the conserved motifs in OBPs and CSPs from *H*. *cunea*, *B*. *mori*, and *H*. *armigera*. The MEME server was used to help us compare motif patterns of OBP and CSP proteins in distinct lepidopteran families. As a result, eight motifs for both OBPs and CSPs were obtained, 27 different motif patterns of 76 OBPs and 25 motif patterns of 43 CSPs. We listed 11 relatively common motif patterns, including 54 OBPs (Fig 7). The most common motif pattern with 14 homologous OBPs (BmorOBP7/8/11/12, HarmOBP1/3/4/6/7, HyphOBP2/18/22/24/25) had a motif order of 4-1-5-3-2; motif 5 and motif 1 were constructed from two motif patterns singly which had seven homologous OBPs (BmorOBP22/28/30, HarmOBP11/18, HyphOBP6/20) and six homologous OBPs (BmorOBP5/13/35/36/39, HyphOBP21), respectively. Interestingly, PBP1 of *H*. *cunea* and *H*. *armigera* had the same motif pattern with the motif order as 6–7; PBP2 of *H*. *cunea* and *H*. *armigera* also had the same motif pattern characterized by motif 2 at the C-terminal with the motif order as 6-7-2, and the motif 6, 7 were only found in the PBPs and located at the same position as the central part.

**Fig 8:**
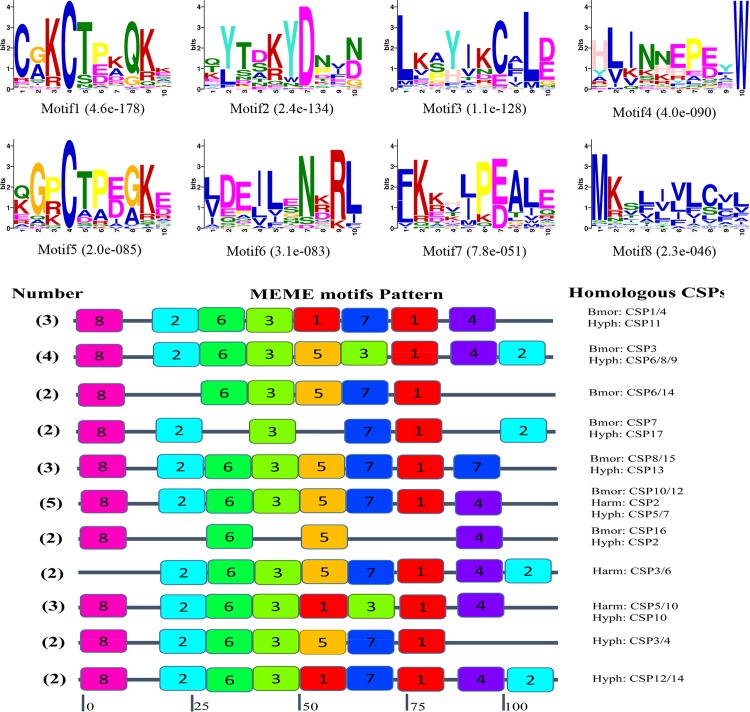
Motif analysis of CSPs in *H*. *cunea*. The upper parts listed the eight motifs discovered in 43 CSPs. The lower parts indicate approximate locations of each motif on the protein sequence. The numbers in the colored boxes correspond to the numbered motifs in the upper part of the figure. The small number represents high conservation. The numbers on the bottom show the approximate locations of each motif on the protein sequence, starting from the N-terminal. This figure just listed 11 relatively common motif patterns including 30 CSPs.

We also list 11 common motif patterns containing 30 CSPs in Fig 8. The motif pattern 8-2-6-3-5-7-1-4 was the only one which had five homologous CSPs (BmorCSP10/12, HarmCSP2, and HyphCSP5/7) from all three species and also the most common pattern. Motif 8 existed in 28 out of 30 CSPs at the N-terminal, with the exception of HarmCSP3/6, and motif 1, 3, which also existed as 28 CSPs with the exception of BmorCSP16 and HyphCSP2 that both located at the central part. In addition, motif 1, 2, 3, 7 existed at different positions infrequently.

### Tissue expression analysis of OBPs and CSPs

**Fig 9:**
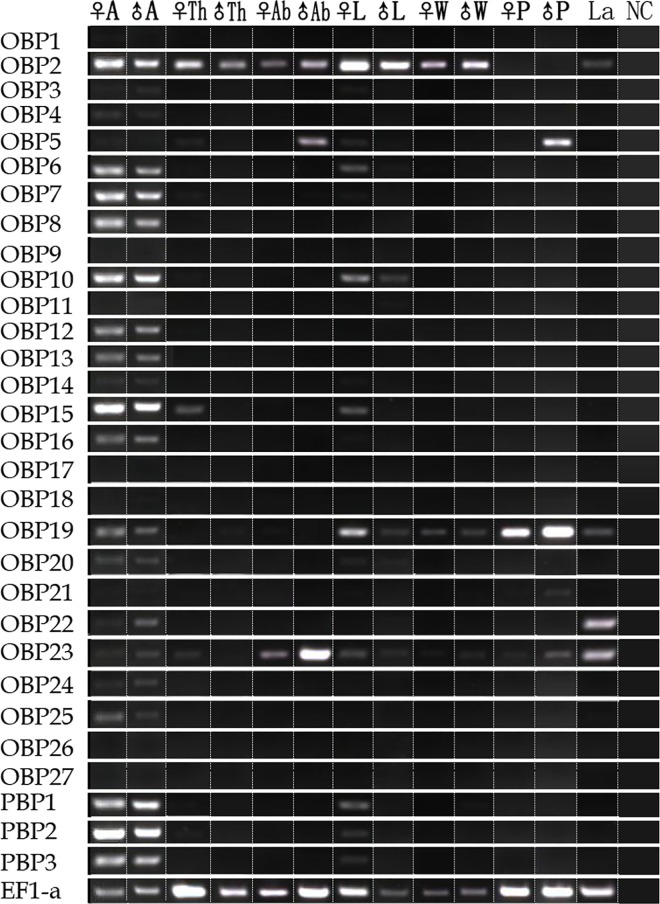
*H*. *cunea* OBPs transcript levels in different tissues and life stages as measured by RT-PCR. A: antennae; Th: thoraces; Ab: abdomens; L: legs; W: wings; P: pupae; La: larvae; NC: no template control; ♀: female; ♂: male. EF1-a was used as a reference gene for each cDNA template.

**Fig 10:**
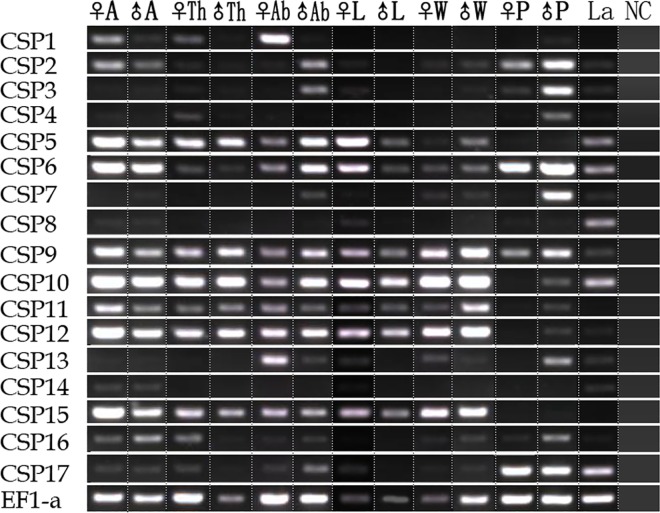
*H*. *cunea* CSPs transcript levels in different tissues and life stages as measured by RT-PCR. A: antennae; Th: thoraces; Ab: abdomens; L: legs; W: wings; P: pupae; La: larvae; NC: no template control; ♀: female; ♂: male. EF1-a was used as a reference gene for each cDNA template.

We analyzed the expression patterns of OBPs and CSPs in different tissues and life stages of *H*. *cunea* using RT-PCR (Figs 9 and 10). The results indicated that 16 OBPs of *H*. *cunea* (*HyphOBP6-8*, *HyphOBP10*, *HyphOBP12-16*, *HyphOBP20*, *HyphOBP22*, *HyphOBP24-25*, and *HyphPBP1-3*) were uniquely or primarily expressed in the female and male antennae. Three OBPs–*HyphOBP2*, *HyphOBP19*, *and HyphOBP23* –were expressed not only in the antennae but also in other tissues like the thoraces, abdomens, legs, and wings, and also in pupae and larvae (Fig 9). As for the CSP genes, 12 CSPs (*HyphCSP1-2*, *HyphCSP5-6*, *HyphCSP9-12* and *HyphCSP14-17*) were relatively intense bands in the female and male antennae. Seven HyphCSP genes (*HyphCSP5-6*, *HyphCSP9-12* and *HyphCSP15*) were expressed in all tested tissues. A wide range of expression in the pupae and larvae of HyphCSP genes (*HyphCSP2-14*, *HyphCSP16-17*) suggested the connection between chemosensory proteins and *H*. *cunea* pupae and larvae, involving various chemosensory processes (Fig 10).

**Fig 11:**
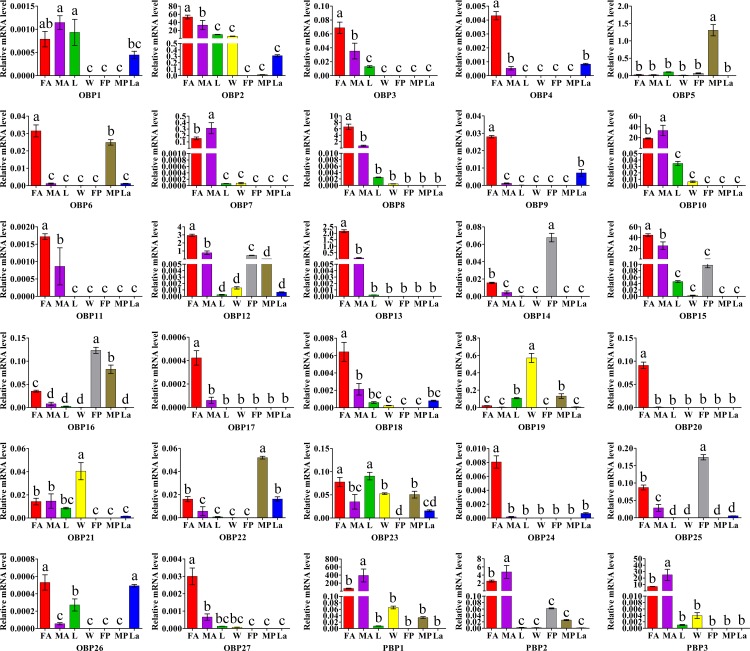
*H*. *cunea* OBPs transcript levels in different tissues and life stages as measured by RT-qPCR. Relative mRNA level in different tissues and life stages were analyzed with Duncan's new multiple range method. The standard errors are represented by error bars, different letters (a, b, c, d) above bars denote significant difference between different tissues and life stages, at the 0.05 level; FA: female antennae; MA: male antennae; L: legs; W: wings; FP: female pupae; MP: male pupae; La: larvae.

**Fig 12:**
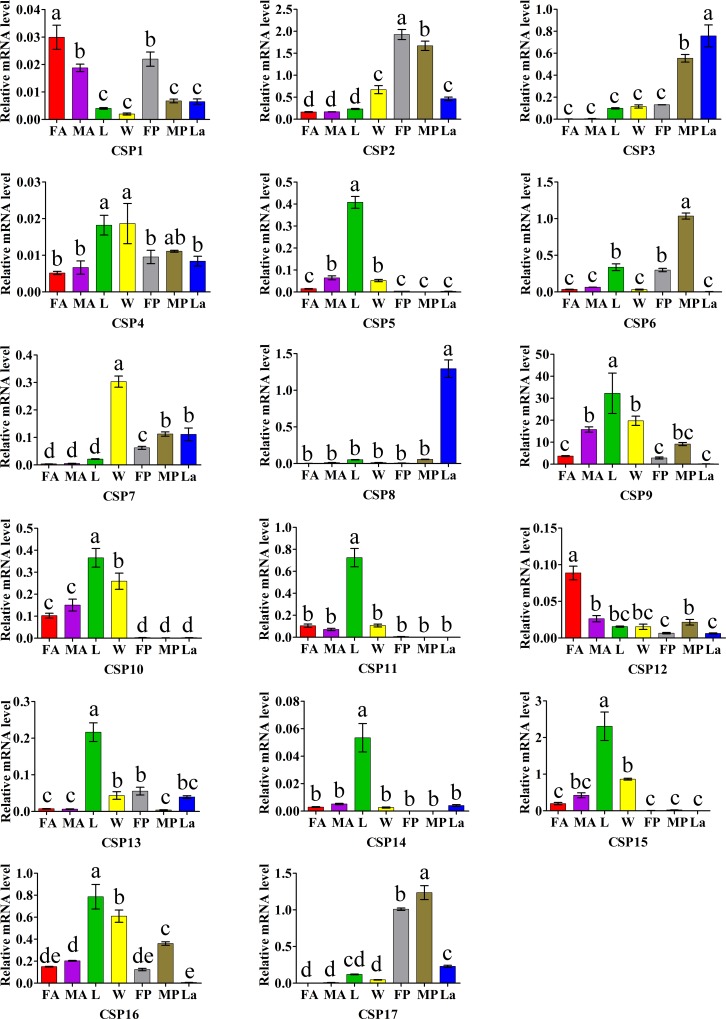
*H*. *cunea* CSPs transcript levels in different tissues and life stages as measured by RT-qPCR. Relative mRNA level in different tissues and life stages were analyzed with Duncan's new multiple range method. The standard errors are represented by error bars, different letters (a, b, c, d) above bars denote significant difference between different tissues and life stages, at the 0.05 level; FA: female antennae; MA: male antennae; L: legs; W: wings; FP: female pupae; MP: male pupae; La: larvae.

In order to confirm the RT-PCR results, real-time quantitative PCR (RT-qPCR) analyses were conducted to characterize the expression profiles of the OBPs and CSPs in different tissues and life stages of *H*. *cunea*. The results showed that all OBPs and CSPs were expressed in antennae, confirming the authenticity of the transcriptome data (Figs 11 and 12). For 22 of the 30 OBPs (including three *HyphPBP*1-3), were observed the highest expression levels in antennae (Fig 11). Two OBPs–*HyphOBP2* and *HyphOBP23*– had a relatively high expression both in antennae and legs. The expression levels of two OBPs (*HyphOBP19* and *HyphOBP21*) in wings were significantly higher than organs. Five OBPs (*HyphOBP5*, *HyphOBP14*, *HyphOBP16*, *HyphOBP22* and *HyphOBP25*) were detected the highest expression levels in pupae and one OBP (*HyphOBP26*) showed a higher expression levels in larvae (Fig 11). In addition, the expression levels of 16 antennae-enriched OBPs (*HyphOBP2-4*, *HyphOBP6*, *HyphOBP8-9*, *HyphOBP11-13*, *HyphOBP15*, *HyphOBP18*, *HyphOBP20*, *HyphOBP23-24*, and *HyphOBP26-27*) was higher in female antennae than in male antennae. Three PBPs (*HyphPBP1-3*) and two OBPs (*HyphOBP7*, and *HyphOBP10*) were significantly overexpressed in male antennae and displayed male antennae-biased expression.

For the CSPs, all of HyphCSP genes were expressed in all tested tissues and life stages. Among of 17 CSPs, eight CSPs (*HyphCSP5*, *HyphCSP9-11*, and *HyphCSP13-16*) and two CSPs (*HyphCSP4* and *HyphCSP7*) were highly enriched in legs and in the wings, respectively. Whereas, only two CSPs (*HyphCSP1* and *HyphCSP12*) showed a significantly higher expression in antennae than in other non-olfactory tissues. In addition, we also found that some CSPs were highly enriched in the pupae and larvae, such as two CSPs (*HyphCSP3* and *HyphCSP8*) in larvae and three CSPs (*HyphCSP2*, *HyphCSP6*, *HyphCSP17*) in pupae (Fig 12).

In the whole, the results from RT-PCR bands were consistent with the results of RT-qPCR. For example, several HyphOBPs (*HyphOBP2*, *HyphOBP10*, *HyphOBP13*, *HyphOBP15*, and *HyphPBP*1-3) and HyphCSPs (*HyphCSP2*, *HyphCSP5-6*, *HyphCSP9-12*, and *HyphCSP15*), which were relatively intense bands in antennae, were also highly enriched in antennae by RT-qPCR (Figs 9–12). In short, the RT-PCR results were verified by RT-qPCR. Whereas, there were a few results of RT-qPCR were to be in disagreement with the bands shown using RT-PCR. For example, *HyphOBP14*, *HyphOBP*16 and *HyphOBP25* were detected in pupae by RT-qPCR, but the expression bands of these genes were very faint by RT-PCR. These differences may owing to the differentiated sensitivity of these two techniques. RT-qPCR is considered to be the most powerful, sensitive, and quantitative assay for the detection of RNA levels.

## Discussion

Numerous olfactory genes have been reported in recent studies of Lepidoptera, such as *H*. *armigera* [48], *M*. *sexta* [2], *Epiphyas postvittana* [27], *Chilo suppressalis* [26], *Cydia pomonella* [24], *D*. *houi* and *D*. *kikuchii* [42]. However, most research has focused on species using Type-I pheromones, with little known about species using the less common Type-II pheromones (Table 8). Here, we have identified numerous olfactory genes from an arctiid moth producing Type-II pheromones, using Illumina Hiseq^TM^ 2500 platform sequencing to analyze the antennal transcriptome of *H*. *cunea* as a step towards understanding olfactory processing in this and related species. In total, 30 OBPs, 17 CSPs, 52 ORs, 14 IRs, 9 GRs, and two SNMPs were identified from the antennae of *H*. *cunea*.

**Table 8:** Summary of olfactory genes and sex pheromone types in Lepidoptera.

Species	Family	Sex pheromone type	OBPs	CSPs	ORs	IRs	GRs	ODEs	SNMPs	Summary
*Helicoverpa armigera*	Noctuidae	TypeI	34	18	60	19	—	—	2	133
*Agrotis ipsilon*	Noctuidae	TypeI	33	12	42	24	1	—	2	114
*Spodoptera litura*	Noctuidae	TypeI	38	18	26	9	—	24	—	115
*Spodoptera littoralis*	Noctuidae	TypeI	36	21	47	17	6	—	—	127
*Spodoptera exigua*	Noctuidae	TypeI	34	20	10	6	6	—	3	79
*Heliothis assulta*	Noctuidae	TypeI	29	17	64	19	—	—	2	131
*Sesamia inferens*	Noctuidae	TypeI	24	24	39	3	—	27	2	119
*Chilo suppressalis*	Pyralidae	TypeI	26	21	47	20	—	—	2	116
*Manduca sexta*	Sphingidae	TypeI	18	21	48	6	1	—	2	96
*Epiphyas postvittana*	Tortricidae	TypeI	34	13	70	19	9	129	2	276
*Hyphantria cunea*	Arctiidae	TypeII	30	17	52	14	9	—	2	124
*Ascotis selenaria cretacea*	Geometridae	TypeII	2	—	—	—	—	—	—	2

In the transcriptome sets, a total of 59,243 unigenes were assembled from 78,131 transcripts. Compared with several reported moth transcriptomes, the mean length of unigenes in *H*. *cunea* (829bp) was longer than *M*. *sexta* (460bp) [2] and *S*.*litura* (603bp) [49], but shorter than *A*. *ipsilon* (967bp) [50] and *H*. *armigera* (991bp) [48]. Furthermore, the minimum length 201bp and the maximum length 29,665bp among all unigenes indicates the high quality and depth of sequencing at the transcriptome level. The species classification obtained by using Blastx in the Nr protein database showed that the highest similarity was to *B*. *mori* (48.1%) and *D*. *plexippus* (29.5%), possibly in part because of the extensive identification of genes, including olfactory genes, from *B*. *mori* (14,623) [46] and *D*. *plexippus* (16866) [51] with the genome sequencing approach. GO and KO annotation were also generated during the bioinformatics analysis. In the *H*. *cunea* transcriptome, 21.1% of the unigenes (12,565) were annotated in GO, slightly more than the KO which comprised 9.75% (5,781), that is, over 70% of the unigenes had no annotation in either the GO or KO databases, suggesting a large number of new potential olfactory genes.

We identified 30 OBPs on the basis of antennal transcriptome of *H*. *cunea* by homology alignment. The number of *H*. *cunea* OBP genes predicted in this study was similar to *H*. *assulta* (29) [52] and *A*. *ipsilon* (33) [50], but less than *S*. *litura* with 38 OBPs [41], or *B*. *mori* (44) [53], the latter of which had whole-genome data. The homology analysis in the phylogenetic tree showed that HyphOBPs were divided into several different branches with another 219 OBPs from nine lepidopteran species, such as the Plus-C subfamily (HyphOBP1, HyphOBP6, HyphOBP23), the Minus-C subfamily (HyphOBP5, HyphOBP19), and the PBP subfamily including HyphPBP1-3. The differential types of *H*. *cunea* OBPs, suggested by the various molecular structures constructed from diverse numbers of cysteines [7], indicates that HyphOBPs may be involved in biological processes other than olfaction. OBPs are a key link in olfactory processing because they transport odorants from the external environment through the sensilla lymph to the ORs [5, 54]. Many researches have shown that insect OBPs are found specifically in antennae [53, 55–57], in our study, most of the OBPs of *H*. *cunea* were highly abundant in the antennae by RT-PCR and RT-qPCR analyses, suggesting their putative role in the odorant detection. However, some OBPs were expressed in tissues other than antennae: *HyphOBP19* and *HyphOBP21* had a relatively high expression in wings than other organs, and *HyphOBP2* and *HyphOBP23* were leg-enriched. In addition, several OBPs (*HyphOBP5*, *HyphOBP14*, *HyphOBP16*, *HyphOBP22*, *HyphOBP25* and *HyphOBP26*) were also enriched in pupae and larvae. These suggest that insect OBPs are widely distributed in other tissues (legs and wings) besides the antennae, and adapt to complex olfaction-related activities in different development stages.

Sex pheromones play a crucial role as signals between sexually reproducing insect species [29]. Moth sex pheromones consist of two major types: Type-I and Type—II. About 75% of known moth sex pheromones are the Type-I, such as those of some species in the Pyralidae, Noctuidae and Tortricidae [19, 20]. Type-II pheromones are found in 15% of lepidopteran species, primarily the Arctiidae and Geometroidea [20, 58]. *H*. *cunea* (Drury) is one of the most destructive species in the group of Arctiidae that use Type-II pheromone. In this study, three PBPs were identified among the 30 OBPs of *H*. *cunea*. According to the RT-qPCR method, all three PBPs (*HyphPBP1-3*) showed high expression level (Fig 11) in antennae and were male antennae-biased, suggesting their putative role in detecting of the female sex pheromones.

Bioinformatics analysis led to the identification of 17 CSPs from our transcriptome data. CSPs are another type of soluble protein that have similar functions to OBPs in carrying semiochemicals [59], while being smaller and more conserved than OBPs. The Blastx analysis of the CSPs proved their relatively high conservation among various species (Table 4). Compared with the unique and/or primary expression in antennae of OBPs, CSPs demonstrated, as many CSPs were found on various body parts such as, antennae, thoraces, abdomens, legs, and wings, even in pupae and larvae (Figs 10 and 12). This ubiquitous expression characteristic of CSPs suggested that they may participate in regulatory mechanisms or other physiological processes in on-olfactory tissues.

In motif pattern analysis by MEME, eight motifs for both 76 OBPs and 43 CSPs from *H*. *cunea*, *B*. *mori*, and *H*. *armigera* were identified. The most common motif pattern in OBPs had a motif order of 4-1-5-3-2, including 14 homologous OBPs and the motif pattern 8-2-6-3-5-7-1-4, which had five homologous CSPs was the most common pattern in CSPs. There still have more conserved motifs in OBPs and CSPs in three distinct lepidopteran families. Similar results also reported by Gu et al and Zhang et al [41, 42], which compared the motif patterns within genus and between Lepidoptera OBPs and CSPs. The most noteworthy is that HyphPBP1 and HyphPBP2 had the same conserved motifs with HarmPBP1 and HarmPBP2, despite the different pheromone types between the two species. Further research on the functional roles of the proteins may explain this phenomenon and determine the binding characteristics of PBPs and the Type-II pheromone components of *H*. *cunea*.

Insect SNMPs are two-transmembrane, olfactory-specific membrane proteins that are homologous with human CD36 receptors [13]. Two SNMPs, SNMP1 and SNMP2, have been identified in insects, and expressed at different locations in antennal sensilla [13, 60–63]. In this study, SNMP1 and SNMP2 were identified, and it is clear that these two SNMPs belong to separate subfamilies from the phylogenetic tree (Fig 6). Nine GRs were discovered in this study, of which, HyphGR3 was clustering with BmorGR8 which has been identified as a sugar receptor; Thus, possibly HyphGR3 plays functions as a sugar receptor, and the antennae of *H*. *cunea* may have a role in sugar detection [64]. This suggests that the antennae of *H*. *cunea* may play a role in sugar detection, and more GRs participating in detection of bitter and other compounds may be found by further study of *H*. *cunea*. We discovered 14 IRs from the antennal transcriptome, and all of them were distributed in different IR subfamilies. Among of them, HyphIR14 and HyphIR12 were identified as the highly conserved coreceptors IR25a, IR8a, respectively, and HyphIR9 was characterized as IR76b subunit, which maybe the second putative coreceptor [65].

ORs are pivotal in sophisticated olfaction systems and have been proposed to be a link between the external environment and insect physiological reactions [5]. A total of 52 ORs were identified in the *H*. *cunea* antennae transcriptome. The number of HyphORs is higher than in other lepidopteran species, such as *H*. *armigera* (47) [48], *S*. *littoralis* (47) [66], *S*. *inferens* (39) [67] and *S*. *litura* (26) [49]. It is generally accepted that ORs are divided into atypical odorant receptors and traditional odorant receptors. In our study, HyphOR27 was identified as one of the atypical odorant receptors, also called ORco, and it clustered with ORco from *B*. *mori* and *S*. *inferens* with >90% homology (Fig 3). Three pheromone receptors (PRs) (HyphOR1, 7, 50) were also located in the PR subcategory branches. HyphOR50 was orthologous to ObruOR1, another known pheromone receptor in species using Type-II pheromone. This phenomenon may indicate the common ancestor of PR genes in Type-II pheromone responding moths. AsegOR3, responding to both Type-I and Type-II pheromones from a Type-I pheromone producing moth, was also clustered with these two PRs, which may suggest similarities in evolution. Several branches were noteworthy, such as-HyphOR39, 33, 3, 25, and 32, which share a high homology with SinfOR19 and forming a separate subset. The same situation occurred with HyphOR40, 29, 45 and SinfOR16. These orthologies suggest similar protein structures and functions between *H*. *cunea* and *S*. *inferens*, which would need to be followed up in further research.

*Chouioia cunea* Yang is a native parasitoid wasp that represents a significant natural enemy to *H*. *cunea* and which could play a vital role in the biological control of the fall webworm [30, 68]. The mechanisms by which *C*. *cunea* locates, recognizes, and parasitizes *H*. *cunea* are not known, but there may be some overlap in the chemosensory abilities of the two species [69]. Thus, we constructed two phylogenetic trees using OBPs and CSPs from *H*. *cunea* and *C*. *cunea* [70] (S7 and S8 Figs). Six clusters (C1, C2, C3, C4, C5, C6) were generated from the OBPs of the two species, indicating some similarities in olfaction between the parasitoid wasp and its host. In particular, HyphOBP10 and CcunOBP13 were considered orthologous, with 75% similarity, and probably similar molecular structure and function [71]. Compared with OBPs, CcunCSP7 had a higher orthology with HyphCSP1 and HyphCSP2, perhaps due to strong conservation of this class of proteins. Similar results have been reported shared OBPs and CSPs from the antennal transcriptome study of another serious pest, *Monochamus alternatus*, and its parasitoid *Dastarcus helpophoroides* [69]. This could be explained by another herbivore-plant-parasitoid system, wherein the homoterpene E-4,8-dimethyl-1,3,7-nonatriene (DMNT), is a key plant compound released by plants under attack by herbivores, and subsequently used as a cue by natural enemies in finding prey; the herbivore species *S*. *littoralis* was in turn deterred by this herbivore-induced plant volatiles [72]. This overlap is ecologically significant, as herbivores and their parasites are expected to share the ability to detect several biological relevant compounds, which may include kairomonal detection of pheromones or herbivore-induced plant volatiles (HIPVs) [69]. Our study provides supporting evidence for the hypothesis that herbivores and their parasites may share olfactory capabilities for perceiving similar biologically relevant compounds. The similarities in proteins may be due to the parasite utilizing similar environmental cues to locate hosts, or possibly for detecting the host directly via *H*. *cunea* pheromones. However, this hypothesis remains to be verified by testing the compounds that could be physiologically or behaviorally active in *H*. *cunea* and *C*. *cunea*. A better understanding of the similarities in chemosensory genes and the interactions between *H*. *cunea* and *C*. *cunea* may indicate an efficient method to eliminate this invasive pest.

## Conclusions

The transcriptome analysis of *H*. *cunea* has provided, for the first time, identification of 124 genes related to the olfactory system of a Type-II lepidopteran pheromone using species and provides insights towards a better understanding of the molecular mechanisms of olfaction for Arctiid moths. Importantly, we found three PBPs (HyphPBP1-3), one putative sugar receptor (HyphGR3), three conserved coreceptors (HyphIR9, HyphIR12 and HyphIR14), one ORco (HyphOR27) and three PRs (HyphOR1, 7, 50), based on phylogenetic analysis. The motifs analysis in OBPs and CSPs from *H*. *cunea*, *B*. *mori*, and *H*. *armigera* were conducted, using a MEME system, and many conserved motif patterns of OBPs and CSPs were found. It was noteworthy that HyphPBP1 and HyphPBP2 had the same conserved motif patterns with HarmPBP1 and HarmPBP2, despite the different pheromone types between the two species. These investigations might provide some insights into the function and evolution of insect OBPs and CSPs. We further verified the expression of OBPs and CSPs by RT-PCR and RT-qPCR analysis and confirmed the authenticity of the transcriptome data. The most of the OBPs had antenna-biased expression and a few of OBPs were enriched in pupae and larvae. And the CSPs demonstrated a ubiquitous expression characteristic. Moreover, three PBPs (*HyphPBP1-3*) were antennae-enriched and displayed a male antennae-biased expression. The tissue and sex-biased expression patterns may provide a deeper further understanding of olfactory processing in *H*. *cunea*. Our work allows for further functional studies of these pheromone binding proteins and potential olfactory receptors in *H*. *cunea*, which may be meaningful targets for the management of this devastating invasive species in China.

## Supporting Information

S1 Fig Distribution of unigene and transcript length interval in the *H*. *cunea* transcriptome assembly. (TIF)

S2 Fig Homology analysis of *H*. *cunea* unigenes. All 15245 unigenes were searched by Blastx against the Nr database with an e-value cut-off of 10^−5^, and analyzed for similarity distribution (A), E-value distribution (B) and species classification (C).
(TIF)

S3 Fig Gene ontology (GO) classification of *H*. *cunea* unigenes. (TIF)

S4 Fig KEGG classification of *H*. *cunea* unigenes. (TIF)

S5 Fig Multiple amino acid sequence alignment of OBPs in *H*. *cunea*. (TIF)

S6 Fig Multiple amino acid sequence alignment of CSPs in *H*. *cunea*. (TIF)

S7 Fig Phylogenetic tree of putative odorant binding protein (OBP) genes from *H*. *cunea* and *C*. *cunea*. Ccun: *C*. *cunea*. (TIF)

S8 Fig Phylogenetic tree of putative chemosensory protein (CSP) genes from *H*. *cunea* and *C*. *cunea*. Ccun: *C*. *cunea*. (TIF)

S1 Table The descriptive statistics and results of ANOVA of OBPs gene expression quantification. (DOCX)

S2 Table The descriptive statistics and results of ANOVA of CSPs gene expression quantification. (DOCX)
