# Correction: Analysis of the Antennal Transcriptome and Insights into Olfactory Genes in *Hyphantria cunea* (Drury)

**DOI:** 10.1371/journal.pone.0235200

**Published:** 2020-06-18

**Authors:** Long-Wa Zhang, Ke Kang, Shi-Chang Jiang, Ya-Nan Zhang, Tian-Tian Wang, Jing Zhang, Long Sun, Yun-Qiu Yang, Chang-Chun Huang, Li-Ya Jiang, De-Gui Ding

The prefixes of all abbreviated names of genes related to *Hyphantria cunea* appear incorrectly throughout the article. The correct gene names are HcunOBPs, HcunPBPs, HcunCSPs, HcunORs, HcunGRs, HcunIRs and HcunSNMPs. Please see the original abbreviations and the corrected abbreviations here.

**Table:** 

**Original abbreviations**	**Corrected abbreviations**
HyphOBPs	HcunOBPs
HyphPBPs	HcunPBPs
HyphCSPs	HcunCSPs
HyphORs	HcunORs
HyphGRs	HcunGRs
HyphIRs	HcunIRs
HyphSNMPs	HcunSNMPs
