# Supplementary figures and images for: Analysis of the Antennal Transcriptome and Insights into Olfactory Genes in Hyphantria cunea (Drury)
Source: PLoS One. 2016 Oct 14;11(10):e0164729. doi: 10.1371/journal.pone.0164729 (PMC5065180; doi:10.1371/journal.pone.0164729)

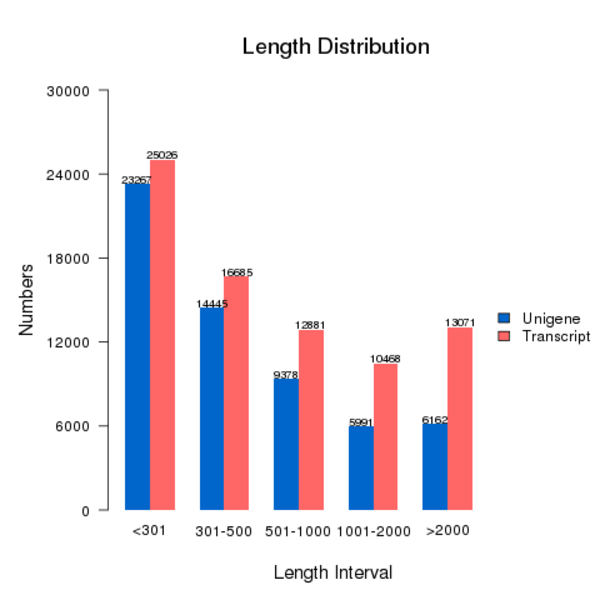

Supplement: S1 Fig — (TIF) [file pone.0164729.s001.tif]

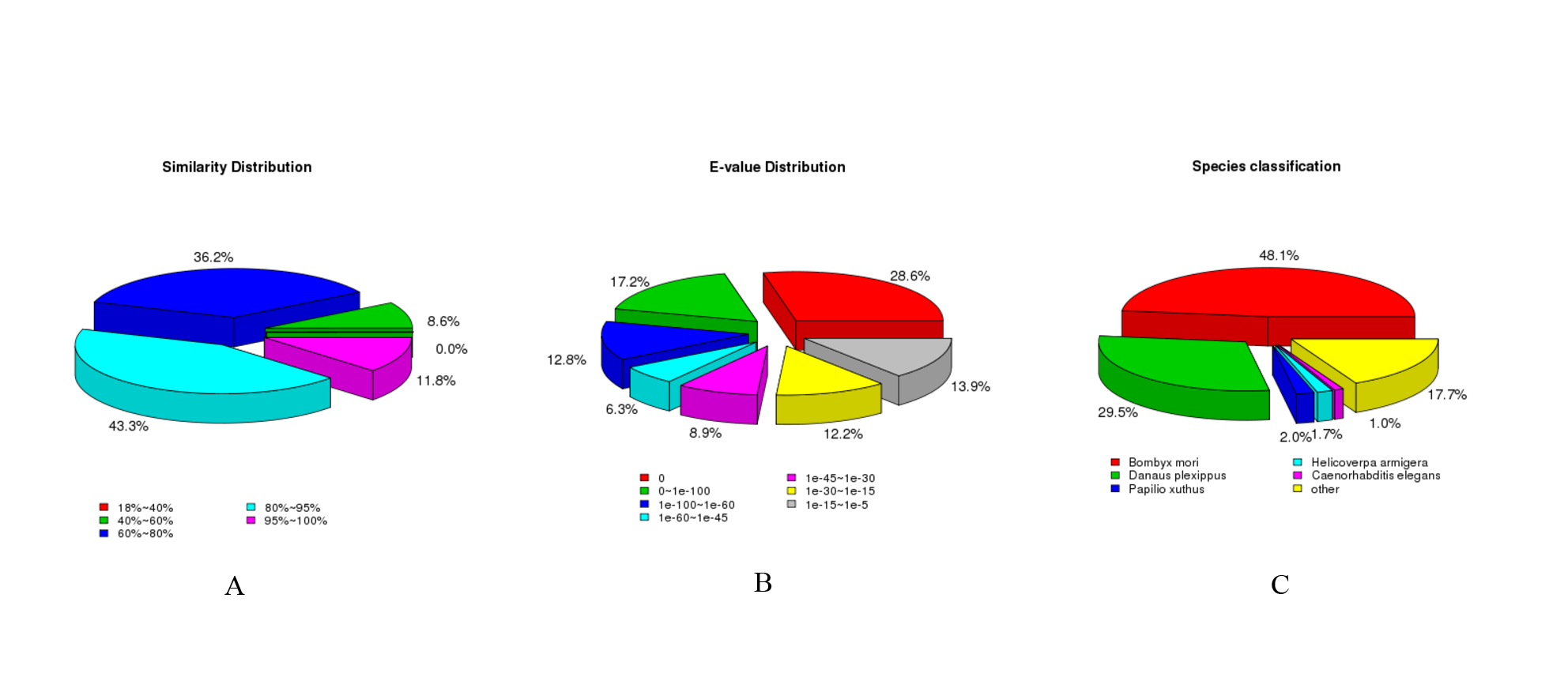

Supplement: S2 Fig — All 15245 unigenes were searched by Blastx against the Nr database with an e-value cut-off of 10−5, and analyzed for similarity distribution (A), E-value distribution (B) and species classification (C). (TIF) [file pone.0164729.s002.tif]

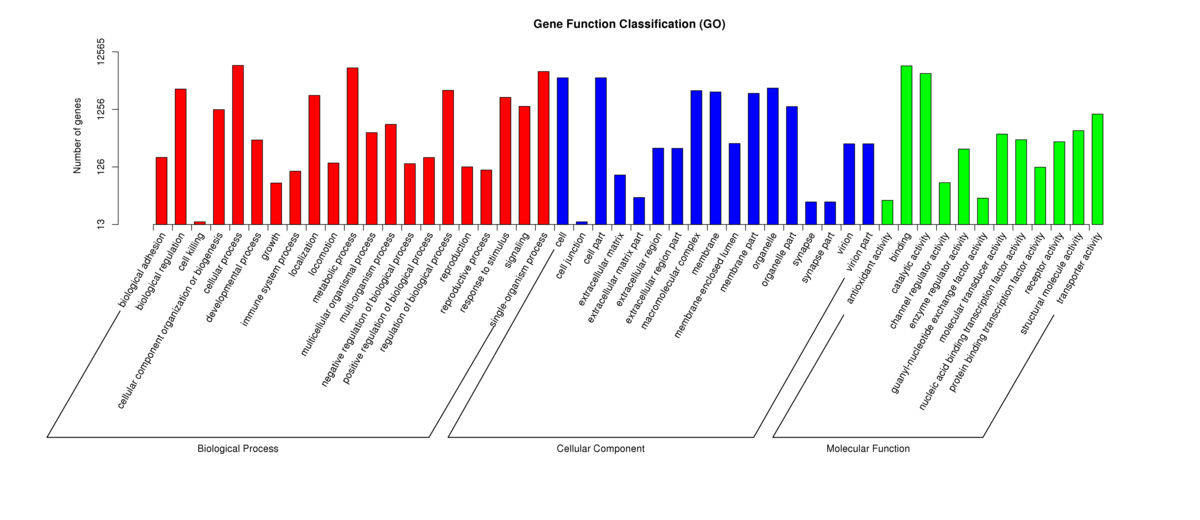

Supplement: S3 Fig — (TIF) [file pone.0164729.s003.tif]

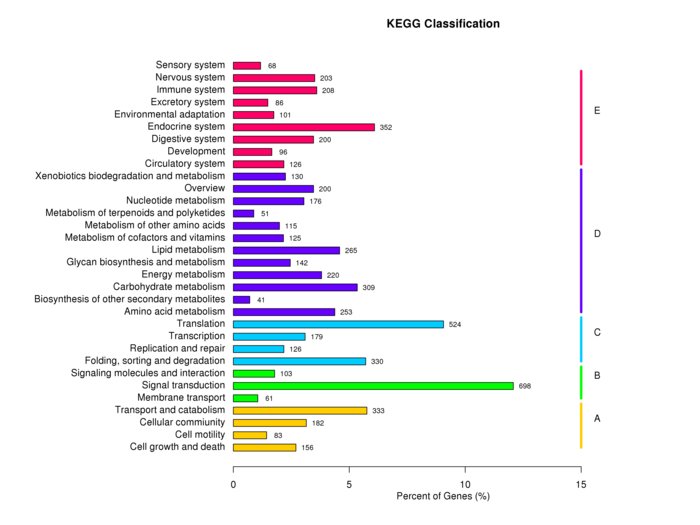

Supplement: S4 Fig — (TIF) [file pone.0164729.s004.tif]

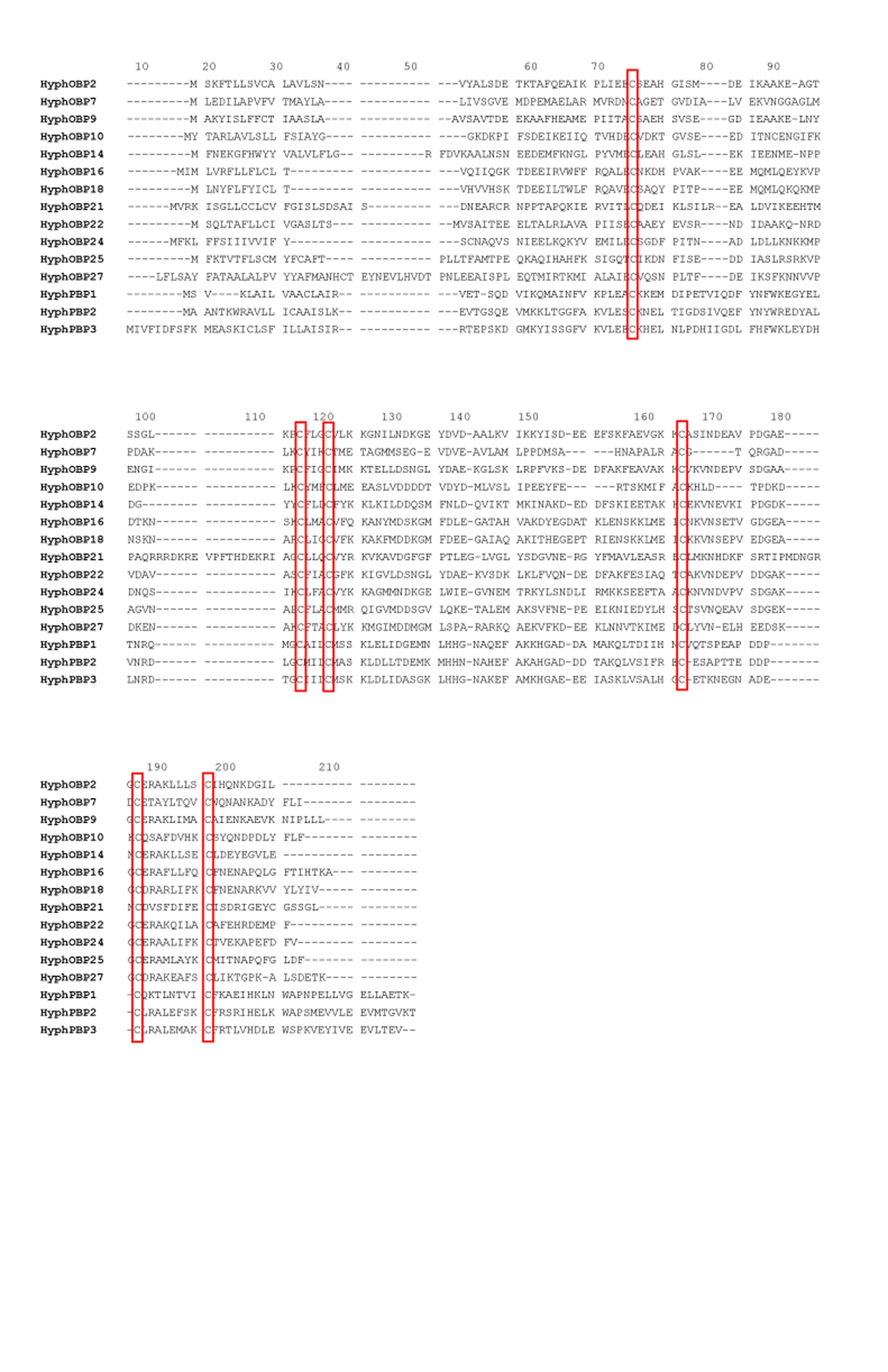

Supplement: S5 Fig — (TIF) [file pone.0164729.s005.tif]

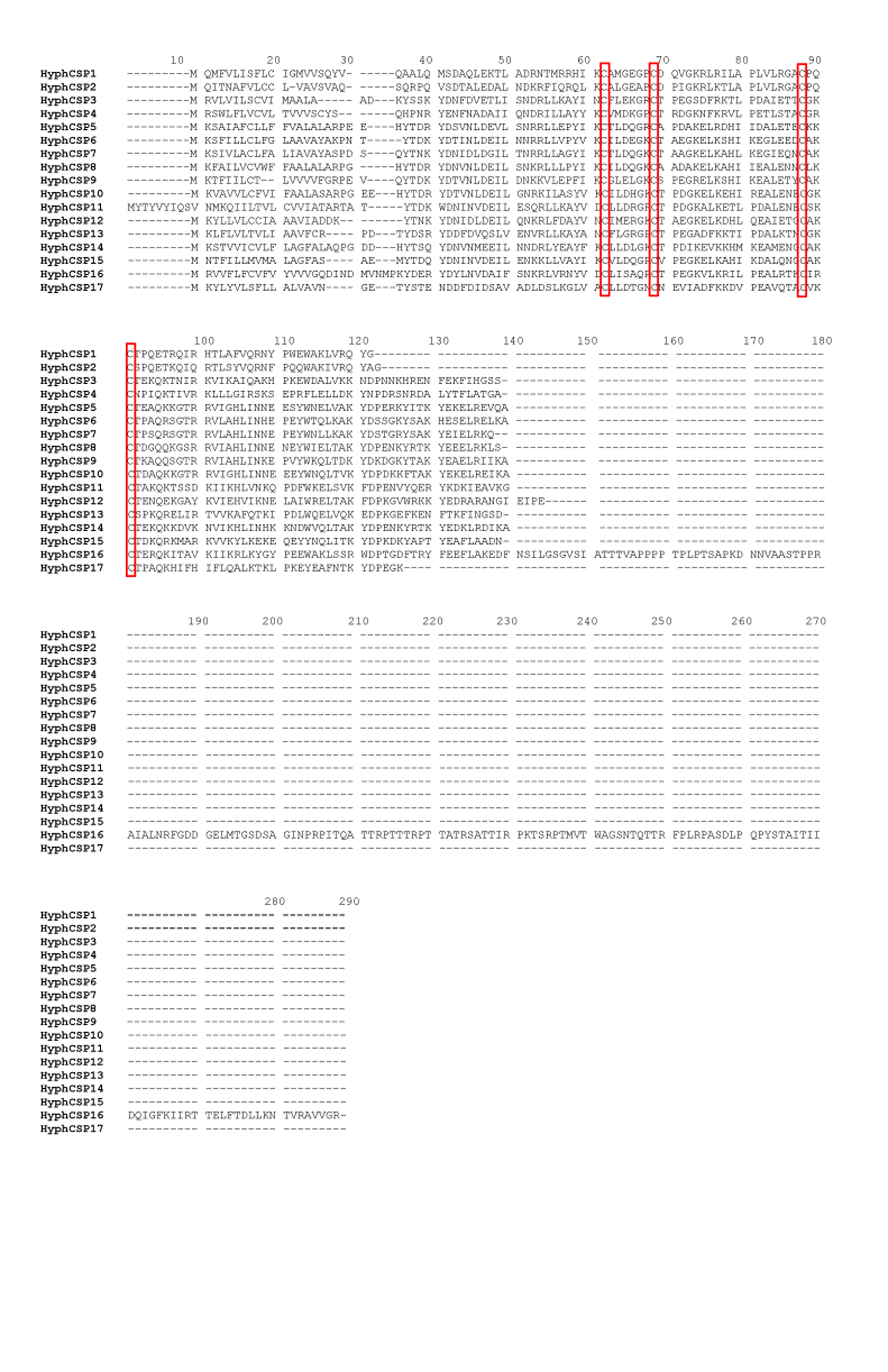

Supplement: S6 Fig — (TIF) [file pone.0164729.s006.tif]

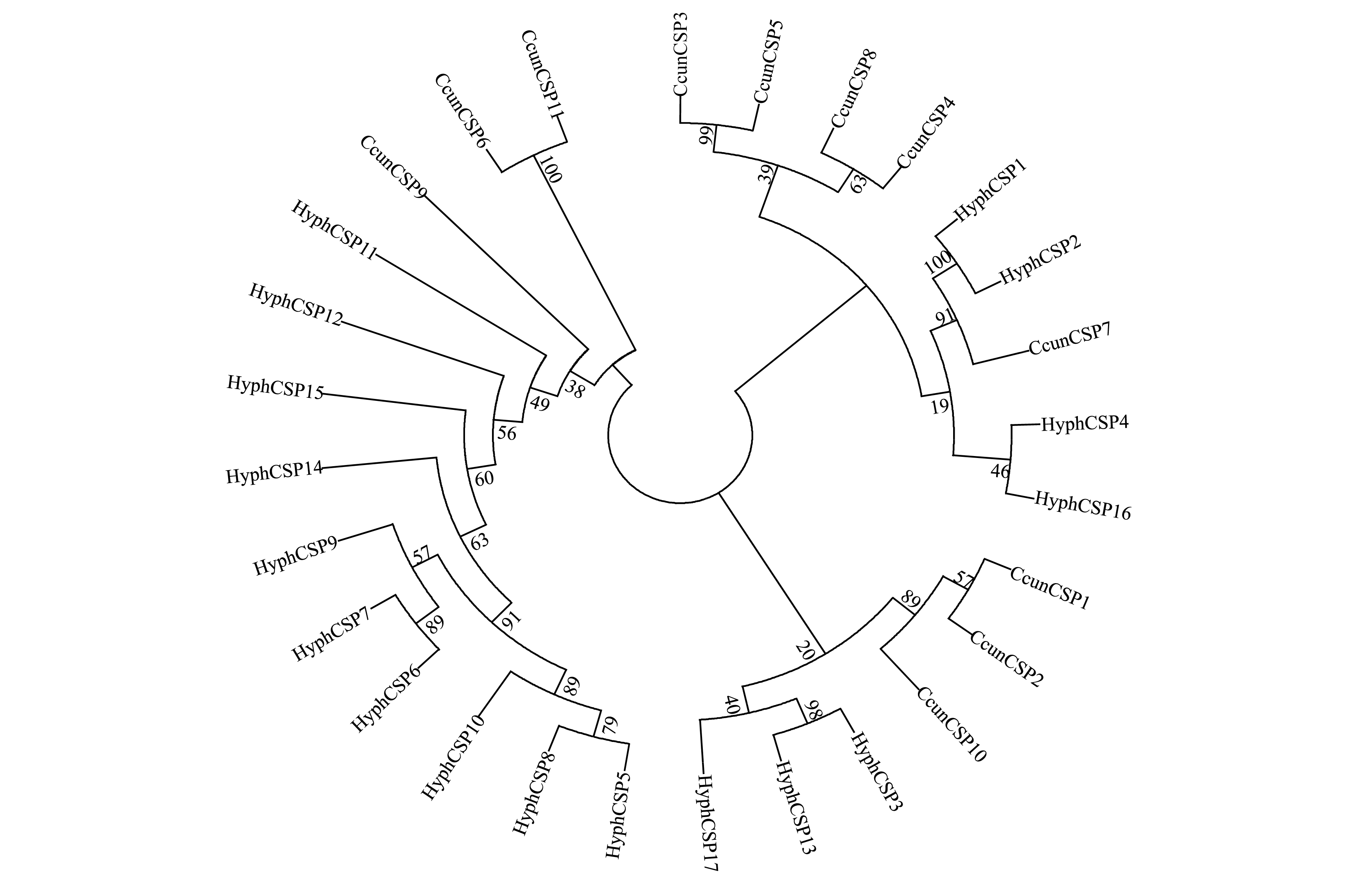

Supplement: S7 Fig — (TIF) [file pone.0164729.s007.tif]

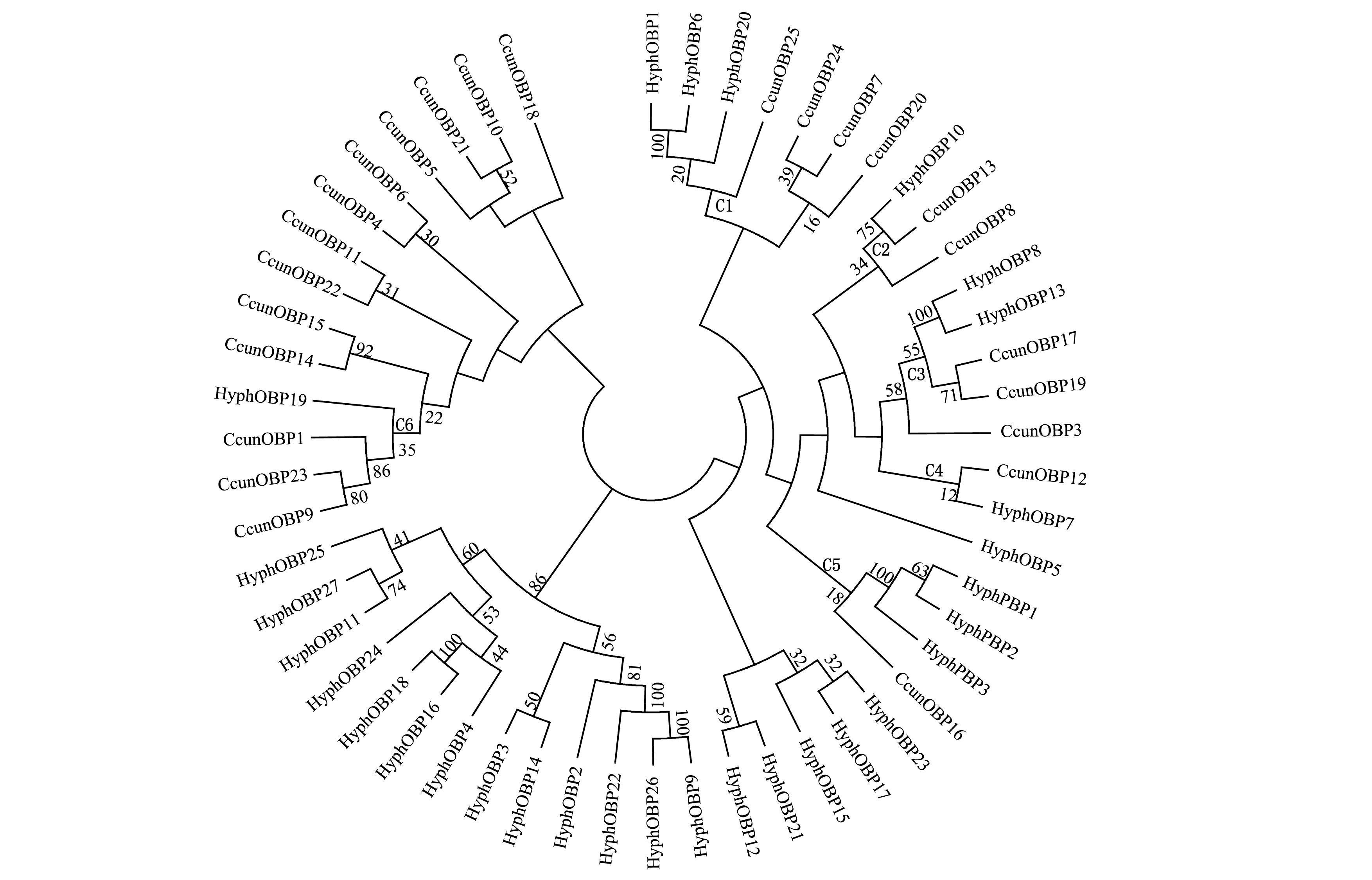

Supplement: S8 Fig — (TIF) [file pone.0164729.s008.tif]
