# Supplementary material for: Analysis of the Antennal Transcriptome and Insights into Olfactory Genes in Hyphantria cunea (Drury)
Source: PLoS One. 2016 Oct 14;11(10):e0164729. doi: 10.1371/journal.pone.0164729 (PMC5065180; doi:10.1371/journal.pone.0164729)
Supplement: S1 Table — (DOCX) [file pone.0164729.s009.docx]

**S1 Table. The descriptive statistics and results of ANOVA of OBPs gene expression quantification.**

| Genes | Tissues | Means | Standard deviation | Standard error | 95% confidence interval | | ANOVA |
| --- | --- | --- | --- | --- | --- | --- | --- |
|  |  |  |  |  | Lower | Upper |  |
| OBP1 | FA | 0.00078773 | 0.000289164 | 0.000166949 | 0.00006941 | 0.00150606 | F(6,14)=12.41  P<0.01 |
|  | MA | 0.00114506 | 0.000255824 | 0.0001477 | 0.00050956 | 0.00178056 |  |
|  | L | 0.00093433 | 0.000486356 | 0.000280798 | -0.00027385 | 0.00214251 |  |
|  | W | 0 | 0 | 0 | 0 | 0 |  |
|  | FP | 0 | 0 | 0 | 0 | 0 |  |
|  | MP | 0 | 0 | 0 | 0 | 0 |  |
|  | La | 0.00044709 | 0.000138487 | 0.000079955 | 0.00010307 | 0.00079111 |  |
| OBP2 | FA | 52.99216141 | 8.187247537 | 4.726909570 | 32.65391105 | 73.33041178 | F(6,14)=19.278  P<0.01 |
|  | MA | 33.13980611 | 19.882777335 | 11.479326847 | -16.25175088 | 82.53136310 |  |
|  | L | 9.69130914 | 0.853328461 | 0.492669417 | 7.57152373 | 11.81109455 |  |
|  | W | 5.42934807 | 0.963330775 | 0.556179282 | 3.03630176 | 7.82239437 |  |
|  | FP | 0 | 0 | 0 | 0 | 0 |  |
|  | MP | 0.01653739 | 0.001299310 | 0.000750157 | 0.01330973 | 0.01976506 |  |
|  | La | 0.31065004 | 0.026776773 | 0.015459577 | 0.24413284 | 0.37716723 |  |
| OBP3 | FA | 0.06882774 | 0.014154239 | 0.008171954 | 0.03366666 | 0.10398882 | F(6,14)=24.973  P<0.01 |
|  | MA | 0.03538738 | 0.019586135 | 0.011308060 | -0.01326728 | 0.08404203 |  |
|  | L | 0.01313441 | 0.001982755 | 0.001144744 | 0.00820898 | 0.01805985 |  |
|  | W | 0 | 0 | 0 | 0 | 0 |  |
|  | FP | 0 | 0 | 0 | 0 | 0 |  |
|  | MP | 0 | 0 | 0 | 0 | 0 |  |
|  | La | 0 | 0 | 0 | 0 | 0 |  |
| OBP4 | FA | 0.00431219 | 0.000516072 | 0.000297954 | 0.00303020 | 0.00559419 | F(6,14)=163.580  P<0.01 |
|  | MA | 0.00052924 | 0.000211086 | 0.000121871 | 0.00000487 | 0.00105361 |  |
|  | L | 0 | 0 | 0 | 0 | 0 |  |
|  | W | 0 | 0 | 0 | 0 | 0 |  |
|  | FP | 0 | 0 | 0 | 0 | 0 |  |
|  | MP | 0 | 0 | 0 | 0 | 0 |  |
|  | La | 0.00082799 | 0.000096904 | 0.000055948 | 0.00058727 | 0.00106872 |  |
| OBP5 | FA | 0.02585660 | 0.015153464 | 0.008748857 | -0.01178670 | 0.06349989 | F(6,14)=60.876  P<0.01 |
|  | MA | 0.02194863 | 0.012613068 | 0.007282158 | -0.00938396 | 0.05328123 |  |
|  | L | 0.10544660 | 0.010459587 | 0.006038845 | 0.07946355 | 0.13142966 |  |
|  | W | 0.01238534 | 0.002007438 | 0.001158995 | 0.00739859 | 0.01737210 |  |
|  | FP | 0.06887126 | 0.019637046 | 0.011337454 | 0.02009014 | 0.11765239 |  |
|  | MP | 1.30561388 | 0.280129577 | 0.161732887 | 0.60973344 | 2.00149433 |  |
|  | La | 0.00601447 | 0.000588214 | 0.000339605 | 0.00455327 | 0.00747568 |  |
| OBP6 | FA | 0.03151470 | 0.005926467 | 0.003421648 | 0.01679254 | 0.04623686 | F(6,14)=91.413  P<0.01 |
|  | MA | 0.00120230 | 0.000561303 | 0.000324068 | -0.00019205 | 0.00259665 |  |
|  | L | 0.00008126 | 0.000020595 | 0.000011890 | 0.00003010 | 0.00013242 |  |
|  | W | 0.00006444 | 0.000014041 | 0.000008107 | 0.00002956 | 0.00009932 |  |
|  | FP | 0 | 0 | 0 | 0 | 0 |  |
|  | MP | 0.02489614 | 0.002712710 | 0.001566184 | 0.01815740 | 0.03163489 |  |
|  | La | 0.00123706 | 0.000193350 | 0.000111631 | 0.00075676 | 0.00171737 |  |
| OBP7 | FA | 0.15466215 | 0.038353546 | 0.022143430 | 0.05938666 | 0.24993764 | F(6,14)=13.375  P<0.01 |
|  | MA | 0.31261746 | 0.148977220 | 0.086012038 | -0.05746248 | 0.68269739 |  |
|  | L | 0.00006762 | 0.000004452 | 0.000002570 | 0.00005656 | 0.00007868 |  |
|  | W | 0.00007819 | 0.000036333 | 0.000020977 | -0.00001207 | 0.00016844 |  |
|  | FP | 0 | 0 | 0 | 0 | 0 |  |
|  | MP | 0 | 0 | 0 | 0 | 0 |  |
|  | La | 0 | 0 | 0 | 0 | 0 |  |
| OBP8 | FA | 6.59733771 | 1.487933024 | 0.859058532 | 2.90110717 | 10.29356825 | F(6,14)=54.592  P<0.01 |
|  | MA | 0.70683205 | 0.345690558 | 0.199584537 | -0.15191090 | 1.56557500 |  |
|  | L | 0.00251300 | 0.000192781 | 0.000111302 | 0.00203411 | 0.00299190 |  |
|  | W | 0.00050902 | 0.000052376 | 0.000030239 | 0.00037891 | 0.00063913 |  |
|  | FP | 0 | 0 | 0 | 0 | 0 |  |
|  | MP | 0 | 0 | 0 | 0 | 0 |  |
|  | La | 0 | 0 | 0 | 0 | 0 |  |
| OBP9 | FA | 0.02802101 | 0.001197897 | 0.000691606 | 0.02504527 | 0.03099675 | F(6,14)=175.468  P<0.01 |
|  | MA | 0.00121633 | 0.000390836 | 0.000225649 | 0.00024544 | 0.00218722 |  |
|  | L | 0.00011465 | 0.000044143 | 0.000025486 | 0.00000499 | 0.00022430 |  |
|  | W | 0 | 0 | 0 | 0 | 0 |  |
|  | FP | 0 | 0 | 0 | 0 | 0 |  |
|  | MP | 0 | 0 | 0 | 0 | 0 |  |
|  | La | 0.00723278 | 0.003366201 | 0.001943477 | -0.00112933 | 0.01559489 |  |
| OBP10 | FA | 18.43339894 | 1.658752410 | 0.957681150 | 14.31282953 | 22.55396836 | F(6,14)=13.127  P<0.01 |
|  | MA | 33.27282620 | 16.763960575 | 9.678677151 | -8.37116047 | 74.91681286 |  |
|  | L | 0.03453016 | 0.005688807 | 0.003284434 | 0.02039838 | 0.04866194 |  |
|  | W | 0.00606783 | 0.002020067 | 0.001166286 | 0.00104970 | 0.01108596 |  |
|  | FP | 0 | 0 | 0 | 0 | 0 |  |
|  | MP | 0 | 0 | 0 | 0 | 0 |  |
|  | La | 0.00038414 | 0.000026569 | 0.000015340 | 0.00031814 | 0.00045014 |  |
| OBP11 | FA | 0.00171716 | 0.000145193 | 0.000083827 | 0.00135648 | 0.00207784 | F(6,14)=10.937  P<0.01 |
|  | MA | 0.00086272 | 0.000925455 | 0.000534312 | -0.00143624 | 0.00316168 |  |
|  | L | 0 | 0 | 0 | 0 | 0 |  |
|  | W | 0 | 0 | 0 | 0 | 0 |  |
|  | FP | 0 | 0 | 0 | 0 | 0 |  |
|  | MP | 0 | 0 | 0 | 0 | 0 |  |
|  | La | 0 | 0 | 0 | 0 | 0 |  |
| OBP12 | FA | 2.94077196 | 0.260288666 | 0.150277732 | 2.29417907 | 3.58736485 | F(6,14)=107.512  P<0.01 |
|  | MA | 0.77667302 | 0.396365611 | 0.228841792 | -0.20795374 | 1.76129978 |  |
|  | L | 0.00025392 | 0.000103663 | 0.000059850 | -0.00000359 | 0.00051144 |  |
|  | W | 0.00132260 | 0.000314525 | 0.000181591 | 0.00054128 | 0.00210393 |  |
|  | FP | 0.45392394 | 0.033015829 | 0.019061698 | 0.37190807 | 0.53593980 |  |
|  | MP | 0.02413296 | 0.002518831 | 0.001454248 | 0.01787584 | 0.03039009 |  |
|  | La | 0.00064069 | 0.000109296 | 0.000063102 | 0.00036918 | 0.00091219 |  |
| OBP13 | FA | 2.16172017 | 0.193684252 | 0.111823655 | 1.68058182 | 2.64285853 | F(6,14)=338.205  P<0.01 |
|  | MA | 0.08560747 | 0.058773160 | 0.033932700 | -0.06039315 | 0.23160809 |  |
|  | L | 0.00022160 | 0.000018640 | 0.000010762 | 0.00017529 | 0.00026790 |  |
|  | W | 0 | 0 | 0 | 0 | 0 |  |
|  | FP | 0 | 0 | 0 | 0 | 0 |  |
|  | MP | 0 | 0 | 0 | 0 | 0 |  |
|  | La | 0 | 0 | 0 | 0 | 0 |  |
| OBP14 | FA | 0.01561679 | 0.001228842 | 0.000709472 | 0.01256418 | 0.01866940 | F(6,14)=159.160  P<0.01 |
|  | MA | 0.00458520 | 0.002895973 | 0.001671991 | -0.00260880 | 0.01177919 |  |
|  | L | 0.00024343 | 0.000045728 | 0.000026401 | 0.00012984 | 0.00035703 |  |
|  | W | 0 | 0 | 0 | 0 | 0 |  |
|  | FP | 0.06768762 | 0.008501681 | 0.004908448 | 0.04656827 | 0.08880696 |  |
|  | MP | 0 | 0 | 0 | 0 | 0 |  |
|  | La | 0 | 0 | 0 | 0 | 0 |  |
| OBP15 | FA | 44.60731131 | 4.269053525 | 2.464739202 | 34.00239446 | 55.21222817 | F(6,14)=38.812  P<0.01 |
|  | MA | 24.78319703 | 12.422116108 | 7.171912079 | -6.07505005 | 55.64144412 |  |
|  | L | 0.04595473 | 0.005285255 | 0.003051443 | 0.03282542 | 0.05908403 |  |
|  | W | 0.00315937 | 0.001385202 | 0.000799747 | -0.00028166 | 0.00660040 |  |
|  | FP | 0.09716867 | 0.014293303 | 0.008252242 | 0.06166214 | 0.13267520 |  |
|  | MP | 0 | 0 | 0 | 0 | 0 |  |
|  | La | 0.00042562 | 0.000049464 | 0.000028558 | 0.00030274 | 0.00054850 |  |
| OBP16 | FA | 0.03534311 | 0.003715578 | 0.002145190 | 0.02611310 | 0.04457312 | F(6,14)=117.649  P<0.01 |
|  | MA | 0.00802509 | 0.006009488 | 0.003469579 | -0.00690330 | 0.02295349 |  |
|  | L | 0.00271046 | 0.000793335 | 0.000458032 | 0.00073971 | 0.00468122 |  |
|  | W | 0.00025728 | 0.000058410 | 0.000033723 | 0.00011218 | 0.00040238 |  |
|  | FP | 0.12340611 | 0.011424278 | 0.006595810 | 0.09502663 | 0.15178559 |  |
|  | MP | 0.08269146 | 0.015598452 | 0.009005770 | 0.04394276 | 0.12144016 |  |
|  | La | 0.00027176 | 0.000056594 | 0.000032674 | 0.00013117 | 0.00041235 |  |
| OBP17 | FA | 0.00042310 | 0.000107009 | 0.000061782 | 0.00015727 | 0.00068892 | F(6,14)=38.336  P<0.01 |
|  | MA | 0.00006018 | 0.000046657 | 0.000026937 | -0.00005572 | 0.00017608 |  |
|  | L | 0 | 0 | 0 | 0 | 0 |  |
|  | W | 0 | 0 | 0 | 0 | 0 |  |
|  | FP | 0 | 0 | 0 | 0 | 0 |  |
|  | MP | 0 | 0 | 0 | 0 | 0 |  |
|  | La | 0 | 0 | 0 | 0 | 0 |  |
| OBP18 | FA | 0.00642808 | 0.001901234 | 0.001097678 | 0.00170515 | 0.01115101 | F(6,14)=22.523  P<0.01 |
|  | MA | 0.00212272 | 0.001147153 | 0.000662309 | -0.00072696 | 0.00497241 |  |
|  | L | 0.00060082 | 0.000170372 | 0.000098365 | 0.00017759 | 0.00102404 |  |
|  | W | 0.00025135 | 0.000040206 | 0.000023213 | 0.00015147 | 0.00035123 |  |
|  | FP | 0 | 0 | 0 | 0 | 0 |  |
|  | MP | 0 | 0 | 0 | 0 | 0 |  |
|  | La | 0.00080099 | 0.000119202 | 0.000068821 | 0.00050488 | 0.00109711 |  |
| OBP19 | FA | 0.02179590 | 0.001862202 | 0.001075143 | 0.01716994 | 0.02642187 | F(6,14)=82.319  P<0.01 |
|  | MA | 0.00397917 | 0.001523275 | 0.000879463 | 0.00019514 | 0.00776319 |  |
|  | L | 0.10828279 | 0.012075787 | 0.006971959 | 0.07828487 | 0.13828071 |  |
|  | W | 0.57084892 | 0.091975186 | 0.053101898 | 0.34236989 | 0.79932795 |  |
|  | FP | 0 | 0 | 0 | 0 | 0 |  |
|  | MP | 0.13184209 | 0.046696187 | 0.026960056 | 0.01584233 | 0.24784185 |  |
|  | La | 0.00638152 | 0.002449181 | 0.001414035 | 0.00029742 | 0.01246562 |  |
| OBP20 | FA | 0.09111387 | 0.012003099 | 0.006929992 | 0.06129652 | 0.12093122 | F(6,14)=171.865  P<0.01 |
|  | MA | 0.00084622 | 0.000570478 | 0.000329365 | -0.00057093 | 0.00226336 |  |
|  | L | 0.00013929 | 0.000034776 | 0.000020078 | 0.00005290 | 0.00022568 |  |
|  | W | 0 | 0 | 0 | 0 | 0 |  |
|  | FP | 0 | 0 | 0 | 0 | 0 |  |
|  | MP | 0 | 0 | 0 | 0 | 0 |  |
|  | La | 0 | 0 | 0 | 0 | 0 |  |
| OBP21 | FA | 0.01421512 | 0.004941340 | 0.002852884 | 0.00194015 | 0.02649009 | F(6,14)=14.036  P<0.01 |
|  | MA | 0.01464165 | 0.010715864 | 0.006186807 | -0.01197804 | 0.04126133 |  |
|  | L | 0.00856434 | 0.001204900 | 0.000695649 | 0.00557121 | 0.01155748 |  |
|  | W | 0.04040701 | 0.012817388 | 0.007400122 | 0.00856685 | 0.07224716 |  |
|  | FP | 0 | 0 | 0 | 0 | 0 |  |
|  | MP | 0 | 0 | 0 | 0 | 0 |  |
|  | La | 0.00163459 | 0.000110562 | 0.000063833 | 0.00135994 | 0.00190924 |  |
| OBP22 | FA | 0.01611397 | 0.003836410 | 0.002214952 | 0.00658380 | 0.02564414 | F(6,14)=96.546  P<0.01 |
|  | MA | 0.00565006 | 0.006722504 | 0.003881240 | -0.01104957 | 0.02234968 |  |
|  | L | 0.00079739 | 0.000546638 | 0.000315601 | -0.00056053 | 0.00215532 |  |
|  | W | 0.00013697 | 0.000040760 | 0.000023533 | 0.00003571 | 0.00023822 |  |
|  | FP | 0 | 0 | 0 | 0 | 0 |  |
|  | MP | 0.05193442 | 0.002096815 | 0.001210596 | 0.04672564 | 0.05714320 |  |
|  | La | 0.01624563 | 0.003249885 | 0.001876322 | 0.00817247 | 0.02431879 |  |
| OBP23 | FA | 0.07770102 | 0.017133870 | 0.009892244 | 0.03513812 | 0.12026391 | F(6,14)=15.388  P<0.01 |
|  | MA | 0.03502377 | 0.027191916 | 0.015699260 | -0.03252470 | 0.10257223 |  |
|  | L | 0.09045083 | 0.014060484 | 0.008117824 | 0.05552265 | 0.12537901 |  |
|  | W | 0.05268543 | 0.002961677 | 0.001709925 | 0.04532822 | 0.06004264 |  |
|  | FP | 0 | 0 | 0 | 0 | 0 |  |
|  | MP | 0.05053484 | 0.012622902 | 0.007287836 | 0.01917781 | 0.08189186 |  |
|  | La | 0.01566028 | 0.003478070 | 0.002008065 | 0.00702028 | 0.02430029 |  |
| OBP24 | FA | 0.00806118 | 0.001532013 | 0.000884508 | 0.00425545 | 0.01186691 | F(6,14)=79.371  P<0.01 |
|  | MA | 0.00017478 | 0.000099115 | 0.000057224 | -0.00007143 | 0.00042100 |  |
|  | L | 0 | 0 | 0 | 0 | 0 |  |
|  | W | 0 | 0 | 0 | 0 | 0 |  |
|  | FP | 0 | 0 | 0 | 0 | 0 |  |
|  | MP | 0 | 0 | 0 | 0 | 0 |  |
|  | La | 0.00066638 | 0.000174117 | 0.000100527 | 0.00023385 | 0.00109891 |  |
| OBP25 | FA | 0.08718063 | 0.012264838 | 0.007081108 | 0.05671309 | 0.11764818 | F(6,14)=148.734  P<0.01 |
|  | MA | 0.02845310 | 0.017279442 | 0.009976290 | -0.01447141 | 0.07137762 |  |
|  | L | 0.00032517 | 0.000134679 | 0.000077757 | -0.00000939 | 0.00065973 |  |
|  | W | 0.00019549 | 0.000031207 | 0.000018017 | 0.00011797 | 0.00027302 |  |
|  | FP | 0.17416818 | 0.012993371 | 0.007501726 | 0.14189085 | 0.20644550 |  |
|  | MP | 0 | 0 | 0 | 0 | 0 |  |
|  | La | 0.00572597 | 0.000146768 | 0.000084736 | 0.00536138 | 0.00609056 |  |
| OBP26 | FA | 0.00052989 | 0.000152095 | 0.000087812 | 0.00015207 | 0.00090772 | F(6,14)=30.575  P<0.01 |
|  | MA | 0.00005480 | 0.000021206 | 0.000012244 | 0.00000212 | 0.00010748 |  |
|  | L | 0.00027080 | 0.000120683 | 0.000069676 | -0.00002899 | 0.00057059 |  |
|  | W | 0 | 0 | 0 | 0 | 0 |  |
|  | FP | 0 | 0 | 0 | 0 | 0 |  |
|  | MP | 0 | 0 | 0 | 0 | 0 |  |
|  | La | 0.00049166 | 0.000026970 | 0.000015571 | 0.00042467 | 0.00055866 |  |
| OBP27 | FA | 0.00300083 | 0.000834105 | 0.000481571 | 0.00092880 | 0.00507287 | F(6,14)=32.313  P<0.01 |
|  | MA | 0.00066018 | 0.000312450 | 0.000180393 | -0.00011599 | 0.00143635 |  |
|  | L | 0.00013183 | 0.000004014 | 0.000002317 | 0.00012186 | 0.00014180 |  |
|  | W | 0.00007574 | 0.000011611 | 0.000006703 | 0.00004690 | 0.00010459 |  |
|  | FP | 0 | 0 | 0 | 0 | 0 |  |
|  | MP | 0 | 0 | 0 | 0 | 0 |  |
|  | La | 0 | 0 | 0 | 0 | 0 |  |
| PBP1 | FA | 65.17746614 | 16.115246500 | 9.304141905 | 25.14497458 | 105.20995771 | F(6,14)=5.695  P<0.01 |
|  | MA | 389.32149170 | 278.123907460 | 160.574912840 | -301.57659536 | 1080.21957876 |  |
|  | L | 0.00779856 | 0.000921203 | 0.000531857 | 0.00551017 | 0.01008695 |  |
|  | W | 0.06541975 | 0.007402551 | 0.004273865 | 0.04703079 | 0.08380870 |  |
|  | FP | 0 | 0 | 0 | 0 | 0 |  |
|  | MP | 0.03396992 | 0.004894973 | 0.002826114 | 0.02181013 | 0.04612971 |  |
|  | La | 0 | 0 | 0 | 0 | 0 |  |
| PBP2 | FA | 2.51348184 | 0.438594003 | 0.253222366 | 1.42395394 | 3.60300974 | F(6,14)=9.779  P<0.01 |
|  | MA | 4.76610663 | 2.724138282 | 1.572781970 | -2.00102801 | 11.53324127 |  |
|  | L | 0.00262853 | 0.000577949 | 0.000333679 | 0.00119283 | 0.00406424 |  |
|  | W | 0.00168031 | 0.000246732 | 0.000142451 | 0.00106739 | 0.00229323 |  |
|  | FP | 0.06196754 | 0.002741629 | 0.001582880 | 0.05515696 | 0.06877812 |  |
|  | MP | 0.02576091 | 0.003149412 | 0.001818314 | 0.01793734 | 0.03358448 |  |
|  | La | 0.00127871 | 0.000237740 | 0.000137260 | 0.00068813 | 0.00186929 |  |
| PBP3 | FA | 6.76398558 | 0.373659628 | 0.215732487 | 5.83576360 | 7.69220755 | F(6,14)=8.139  P<0.01 |
|  | MA | 24.98443651 | 15.034177707 | 8.679986546 | -12.36253130 | 62.33140431 |  |
|  | L | 0.00103541 | 0.000235618 | 0.000136034 | 0.00045011 | 0.00162072 |  |
|  | W | 0.00395616 | 0.001568080 | 0.000905331 | 0.00006084 | 0.00785149 |  |
|  | FP | 0 | 0 | 0 | 0 | 0 |  |
|  | MP | 0 | 0 | 0 | 0 | 0 |  |
|  | La | 0 | 0 | 0 | 0 | 0 |  |
