# Supplementary material for: Analysis of the Antennal Transcriptome and Insights into Olfactory Genes in Hyphantria cunea (Drury)
Source: PLoS One. 2016 Oct 14;11(10):e0164729. doi: 10.1371/journal.pone.0164729 (PMC5065180; doi:10.1371/journal.pone.0164729)
Supplement: S2 Table — (DOCX) [file pone.0164729.s010.docx]

**S2 Table. The descriptive statistics and results of ANOVA of CSPs gene expression quantification.**

| Genes | Tissues | Means | Standard deviation | Standard error | 95% confidence interval | | ANOVA |
| --- | --- | --- | --- | --- | --- | --- | --- |
|  |  |  |  |  | Lower | Upper |  |
| CSP1 | FA | 0.02992152 | 0.007625024 | 0.004402310 | 0.01097991 | 0.04886312 | F(6,14)=26.998  P<0.01 |
|  | MA | 0.01879033 | 0.002359958 | 0.001362522 | 0.01292787 | 0.02465279 |  |
|  | L | 0.00401634 | 0.000514516 | 0.000297056 | 0.00273821 | 0.00529447 |  |
|  | W | 0.00198239 | 0.000639387 | 0.000369150 | 0.00039407 | 0.00357072 |  |
|  | FP | 0.02197535 | 0.004462441 | 0.002576392 | 0.01089003 | 0.03306067 |  |
|  | MP | 0.00673676 | 0.001186644 | 0.000685109 | 0.00378897 | 0.00968454 |  |
|  | La | 0.00647578 | 0.001727881 | 0.000997593 | 0.00218349 | 0.01076808 |  |
| CSP2 | FA | 0.16323425 | 0.016541465 | 0.009550219 | 0.12214297 | 0.20432552 | F(6,14)=110.737  P<0.01 |
|  | MA | 0.16799585 | 0.003939416 | 0.002274423 | 0.15820980 | 0.17778190 |  |
|  | L | 0.23235091 | 0.022764148 | 0.013142887 | 0.17580163 | 0.28890019 |  |
|  | W | 0.67212181 | 0.157971839 | 0.091205084 | 0.27969801 | 1.06454561 |  |
|  | FP | 1.92642507 | 0.198645529 | 0.114688049 | 1.43296222 | 2.41988792 |  |
|  | MP | 1.66947734 | 0.183476985 | 0.105930487 | 1.21369524 | 2.12525944 |  |
|  | La | 0.46504026 | 0.066202436 | 0.038221994 | 0.30058429 | 0.62949623 |  |
| CSP3 | FA | 0.00200997 | 0.001226545 | 0.000708146 | -0.00103694 | 0.00505687 | F(6,14)=54.065  P<0.01 |
|  | MA | 0.00482654 | 0.002044881 | 0.001180613 | -0.00025323 | 0.00990631 |  |
|  | L | 0.09890522 | 0.010324026 | 0.005960579 | 0.07325892 | 0.12455152 |  |
|  | W | 0.11568242 | 0.025169695 | 0.014531730 | 0.05315744 | 0.17820741 |  |
|  | FP | 0.13110435 | 0.001138263 | 0.000657176 | 0.12827675 | 0.13393195 |  |
|  | MP | 0.55432065 | 0.059523428 | 0.034365867 | 0.40645625 | 0.70218504 |  |
|  | La | 0.75824063 | 0.172610413 | 0.099656669 | 0.32945259 | 1.18702867 |  |
| CSP4 | FA | 0.00516545 | 0.000755935 | 0.000436440 | 0.00328760 | 0.00704329 | F(6,14)=4.378  P<0.01 |
|  | MA | 0.00665271 | 0.003122714 | 0.001802900 | -0.00110454 | 0.01440996 |  |
|  | L | 0.01821470 | 0.004651947 | 0.002685803 | 0.00665863 | 0.02977078 |  |
|  | W | 0.01865103 | 0.009498928 | 0.005484209 | -0.00494562 | 0.04224767 |  |
|  | FP | 0.00954367 | 0.003160141 | 0.001824508 | 0.00169344 | 0.01739389 |  |
|  | MP | 0.01110126 | 0.000461015 | 0.000266167 | 0.00995603 | 0.01224648 |  |
|  | La | 0.00839992 | 0.002321872 | 0.001340534 | 0.00263207 | 0.01416777 |  |
| CSP5 | FA | 0.01467756 | 0.001892022 | 0.001092360 | 0.00997752 | 0.01937760 | F(6,14)=184.458  P<0.01 |
|  | MA | 0.06454084 | 0.015345151 | 0.008859527 | 0.02642138 | 0.10266031 |  |
|  | L | 0.40802089 | 0.046468486 | 0.026828593 | 0.29258677 | 0.52345500 |  |
|  | W | 0.05214948 | 0.008957910 | 0.005171852 | 0.02989680 | 0.07440216 |  |
|  | FP | 0.00436197 | 0.000101740 | 0.000058740 | 0.00410923 | 0.00461470 |  |
|  | MP | 0.00141154 | 0.000105293 | 0.000060791 | 0.00114998 | 0.00167310 |  |
|  | La | 0.00328450 | 0.000500139 | 0.000288755 | 0.00204209 | 0.00452692 |  |
| CSP6 | FA | 0.03418291 | 0.004850677 | 0.002800540 | 0.02213316 | 0.04623266 | F(6,14)=215.066  P<0.01 |
|  | MA | 0.06474805 | 0.001460774 | 0.000843378 | 0.06111929 | 0.06837681 |  |
|  | L | 0.33674595 | 0.079653261 | 0.045987832 | 0.13887628 | 0.53461563 |  |
|  | W | 0.03225938 | 0.010878468 | 0.006280686 | 0.00523577 | 0.05928299 |  |
|  | FP | 0.29853344 | 0.040414687 | 0.023333430 | 0.19813779 | 0.39892909 |  |
|  | MP | 1.03595196 | 0.071769266 | 0.041436005 | 0.85766722 | 1.21423670 |  |
|  | La | 0.00469045 | 0.000786794 | 0.000454256 | 0.00273594 | 0.00664495 |  |
| CSP7 | FA | 0.00367703 | 0.000540330 | 0.000311960 | 0.00233477 | 0.00501928 | F(6,14)=74.602  P<0.01 |
|  | MA | 0.00525887 | 0.001229590 | 0.000709904 | 0.00220440 | 0.00831334 |  |
|  | L | 0.02115101 | 0.002548378 | 0.001471307 | 0.01482049 | 0.02748153 |  |
|  | W | 0.30324606 | 0.035368879 | 0.020420232 | 0.21538489 | 0.39110723 |  |
|  | FP | 0.06209794 | 0.009540979 | 0.005508487 | 0.03839683 | 0.08579904 |  |
|  | MP | 0.11236999 | 0.013513470 | 0.007802006 | 0.07880067 | 0.14593932 |  |
|  | La | 0.11125845 | 0.039872800 | 0.023020572 | 0.01220892 | 0.21030797 |  |
| CSP8 | FA | 0.00241661 | 0.000797888 | 0.000460661 | 0.00043454 | 0.00439867 | F(6,14)=112.048  P<0.01 |
|  | MA | 0.01184042 | 0.003705318 | 0.002139266 | 0.00263590 | 0.02104494 |  |
|  | L | 0.05146109 | 0.004327605 | 0.002498544 | 0.04071072 | 0.06221146 |  |
|  | W | 0.01189769 | 0.004839127 | 0.002793871 | -0.00012337 | 0.02391875 |  |
|  | FP | 0.00702352 | 0.001055246 | 0.000609246 | 0.00440214 | 0.00964489 |  |
|  | MP | 0.05671665 | 0.004310077 | 0.002488424 | 0.04600983 | 0.06742348 |  |
|  | La | 1.29463566 | 0.208016631 | 0.120098458 | 0.77789370 | 1.81137761 |  |
| CSP9 | FA | 3.67749443 | 0.372326842 | 0.214963002 | 2.75258328 | 4.60240558 | F(6,14)=10.040  P<0.01 |
|  | MA | 15.77317104 | 2.121114059 | 1.224625773 | 10.50403162 | 21.04231047 |  |
|  | L | 32.24321819 | 15.929139325 | 9.196692877 | -7.32695752 | 71.81339390 |  |
|  | W | 19.73592735 | 3.642188682 | 2.102818616 | 10.68822909 | 28.78362561 |  |
|  | FP | 2.80559407 | 0.810484504 | 0.467933446 | 0.79223895 | 4.81894919 |  |
|  | MP | 9.16151242 | 1.225136115 | 0.707332666 | 6.11810559 | 12.20491924 |  |
|  | La | 0.17915262 | 0.031555132 | 0.018218364 | 0.10076533 | 0.25753992 |  |
| CSP10 | FA | 0.10254726 | 0.019339305 | 0.011165553 | 0.05450576 | 0.15058876 | F(6,14)=35.584  P<0.01 |
|  | MA | 0.15045673 | 0.046936902 | 0.027099033 | 0.03385900 | 0.26705446 |  |
|  | L | 0.36572855 | 0.073517429 | 0.042445308 | 0.18310113 | 0.54835597 |  |
|  | W | 0.25924607 | 0.063669717 | 0.036759728 | 0.10108172 | 0.41741041 |  |
|  | FP | 0.00271798 | 0.000363432 | 0.000209827 | 0.00181516 | 0.00362079 |  |
|  | MP | 0.00155468 | 0.000254621 | 0.000147005 | 0.00092216 | 0.00218719 |  |
|  | La | 0.00223483 | 0.000766651 | 0.000442626 | 0.00033036 | 0.00413930 |  |
| CSP11 | FA | 0.10483504 | 0.026657587 | 0.015390765 | 0.03861392 | 0.17105616 | F(6,14)=63.554  P<0.01 |
|  | MA | 0.07087893 | 0.018196799 | 0.010505927 | 0.02567558 | 0.11608228 |  |
|  | L | 0.72439131 | 0.144017356 | 0.083148459 | 0.36663236 | 1.08215025 |  |
|  | W | 0.10683541 | 0.022233770 | 0.012836673 | 0.05160367 | 0.16206716 |  |
|  | FP | 0.00632528 | 0.001380545 | 0.000797058 | 0.00289581 | 0.00975474 |  |
|  | MP | 0.00120263 | 0.000455079 | 0.000262740 | 0.00007215 | 0.00233311 |  |
|  | La | 0.00221268 | 0.000297455 | 0.000171736 | 0.00147376 | 0.00295161 |  |
| CSP12 | FA | 0.08877759 | 0.016132242 | 0.009313954 | 0.04870288 | 0.12885230 | F(6,14)=42.949  P<0.01 |
|  | MA | 0.02626915 | 0.007344442 | 0.004240316 | 0.00802454 | 0.04451375 |  |
|  | L | 0.01552358 | 0.001403422 | 0.000810266 | 0.01203729 | 0.01900988 |  |
|  | W | 0.01533112 | 0.006251744 | 0.003609446 | -0.00019907 | 0.03086132 |  |
|  | FP | 0.00656704 | 0.001778655 | 0.001026907 | 0.00214862 | 0.01098547 |  |
|  | MP | 0.02155200 | 0.006608615 | 0.003815485 | 0.00513529 | 0.03796870 |  |
|  | La | 0.00619053 | 0.001277838 | 0.000737760 | 0.00301621 | 0.00936485 |  |
| CSP13 | FA | 0.00726163 | 0.000999925 | 0.000577307 | 0.00477767 | 0.00974558 | F(6,14)=44.082  P<0.01 |
|  | MA | 0.00627969 | 0.001267726 | 0.000731922 | 0.00313049 | 0.00942890 |  |
|  | L | 0.21638463 | 0.044313747 | 0.025584554 | 0.10630318 | 0.32646608 |  |
|  | W | 0.04349401 | 0.018141239 | 0.010473849 | -0.00157133 | 0.08855935 |  |
|  | FP | 0.05547993 | 0.018318435 | 0.010576153 | 0.00997441 | 0.10098544 |  |
|  | MP | 0.00385047 | 0.001164138 | 0.000672116 | 0.00095859 | 0.00674235 |  |
|  | La | 0.03927116 | 0.006401505 | 0.003695911 | 0.02336893 | 0.05517338 |  |
| CSP14 | FA | 0.00291100 | 0.000531438 | 0.000306826 | 0.00159083 | 0.00423116 | F(6,14)=24.411  P<0.01 |
|  | MA | 0.00516407 | 0.000866092 | 0.000500038 | 0.00301258 | 0.00731556 |  |
|  | L | 0.05335713 | 0.017859257 | 0.010311047 | 0.00899228 | 0.09772199 |  |
|  | W | 0.00253692 | 0.000696523 | 0.000402137 | 0.00080666 | 0.00426717 |  |
|  | FP | 0 | 0 | 0 | 0 | 0 |  |
|  | MP | 0 | 0 | 0 | 0 | 0 |  |
|  | La | 0.00403371 | 0.001141535 | 0.000659066 | 0.00119798 | 0.00686944 |  |
| CSP15 | FA | 0.19660468 | 0.053835329 | 0.031081842 | 0.06287031 | 0.33033905 | F(6,14)=31.162  P<0.01 |
|  | MA | 0.42314785 | 0.117390176 | 0.067775250 | 0.13153448 | 0.71476121 |  |
|  | L | 2.30700400 | 0.672760622 | 0.388418526 | 0.63577397 | 3.97823403 |  |
|  | W | 0.86325670 | 0.043460528 | 0.025091948 | 0.75529477 | 0.97121864 |  |
|  | FP | 0.00785285 | 0.001114460 | 0.000643433 | 0.00508438 | 0.01062132 |  |
|  | MP | 0.02394641 | 0.003070535 | 0.001772774 | 0.01631878 | 0.03157404 |  |
|  | La | 0.00366221 | 0.000971691 | 0.000561006 | 0.00124839 | 0.00607602 |  |
| CSP16 | FA | 0.15005149 | 0.005159769 | 0.002978994 | 0.13723391 | 0.16286907 | F(6,14)=35.354  P<0.01 |
|  | MA | 0.20333007 | 0.006784492 | 0.003917028 | 0.18647646 | 0.22018368 |  |
|  | L | 0.78634307 | 0.193016566 | 0.111438166 | 0.30686334 | 1.26582280 |  |
|  | W | 0.60973465 | 0.096710530 | 0.055835851 | 0.36949237 | 0.84997692 |  |
|  | FP | 0.12375218 | 0.018261520 | 0.010543293 | 0.07838805 | 0.16911631 |  |
|  | MP | 0.36015727 | 0.027659062 | 0.015968967 | 0.29144835 | 0.42886619 |  |
|  | La | 0.00595482 | 0.001919282 | 0.001108098 | 0.00118706 | 0.01072258 |  |
| CSP17 | FA | 0.00328578 | 0.000614545 | 0.000354808 | 0.00175917 | 0.00481240 | F(6,14)=196.435  P<0.01 |
|  | MA | 0.00830668 | 0.002118599 | 0.001223174 | 0.00304378 | 0.01356957 |  |
|  | L | 0.11904982 | 0.008595553 | 0.004962645 | 0.09769728 | 0.14040235 |  |
|  | W | 0.04550442 | 0.005406131 | 0.003121231 | 0.03207485 | 0.05893400 |  |
|  | FP | 1.00976275 | 0.028554750 | 0.016486093 | 0.93882881 | 1.08069668 |  |
|  | MP | 1.23584569 | 0.164582929 | 0.095021998 | 0.82699903 | 1.64469235 |  |
|  | La | 0.22957640 | 0.026579350 | 0.015345595 | 0.16354963 | 0.29560317 |  |
